# Comparative Proteomic Analysis of Psychrophilic vs. Mesophilic Bacterial Species Reveals Different Strategies to Achieve Temperature Adaptation

**DOI:** 10.3389/fmicb.2022.841359

**Published:** 2022-05-03

**Authors:** Laura García-Descalzo, Eva García-López, Cristina Cid

**Affiliations:** ^1^Centro de Astrobiología, Department of Planetology and Habitability, CSIC-INTA, Madrid, Spain; ^2^Centro de Astrobiología, Department of Molecular Ecology, CSIC-INTA, Madrid, Spain

**Keywords:** psychrophiles, proteomics, adaptation, chaperone protein, protein complex

## Abstract

The old debate of nature (genes) vs. nurture (environmental variables) is once again topical concerning the effect of climate change on environmental microorganisms. Specifically, the Polar Regions are experiencing a drastic increase in temperature caused by the rise in greenhouse gas emissions. This study, in an attempt to mimic the molecular adaptation of polar microorganisms, combines proteomic approaches with a classical microbiological analysis in three bacterial species *Shewanella oneidensis*, *Shewanella frigidimarina*, and *Psychrobacter frigidicola*. Both shewanellas are members of the same genus but they live in different environments. On the other hand, *Shewanella frigidimarina* and *Psychrobacter frigidicola* share the same natural environment but belong to a different genus. The comparison of the strategies employed by each bacterial species estimates the contribution of genome vs. environmental variables in the adaptation to temperature. The results show a greater versatility of acclimatization for the genus *Shewanella* with respect to *Psychrobacter*. Besides, *S. frigidimarina* was the best-adapted species to thermal variations in the temperature range 4–30°C and displayed several adaptation mechanisms common with the other two species. Regarding the molecular machinery used by these bacteria to face the consequences of temperature changes, chaperones have a pivoting role. They form complexes with other proteins in the response to the environment, establishing cooperation with transmembrane proteins, elongation factors, and proteins for protection against oxidative damage.

## Introduction

Cold is the most frequently spread natural condition that causes stress to live organisms (Rodrigues and Tiedje, 2008). Cold ecosystems are widespread on Earth, given that 80% of the biosphere is permanently frozen (Russell, 1990). About 90% of oceans hold a temperature of ≤5°C (Rodrigues and Tiedje, 2008), and, along with the seas, constitute 70% of the surface of the planet with a medium temperature of 2°C.

The low temperature causes changes in the properties of water even if it does not freeze (Rodrigues and Tiedje, 2008). This restricts its availability for biochemical reactions, limiting the survival of microorganisms that are unable to develop adaptive mechanisms to cope with it. When the temperature decreases, the diffusion of water drops, and its viscosity increases, reducing substrate diffusion rates and disrupting the transport of nutrients in the cells (Rodrigues and Tiedje, 2008). However, the low temperature does also alter other important parameters in cells, such as enzyme activity, membrane fluidity, transcription, translation, cellular division rates, protein folding, and denaturation (Russell, 1990; D’Amico et al., 2006; Rodrigues and Tiedje, 2008; Schumann, 2009; Alcazar et al., 2010).

The study of extremophiles has increased the discovery of new microorganisms and has contributed to understanding how they respond to evolving challenges over time at the molecular level (Irwin and Baird, 2004). Regarding the temperature of growth, microorganisms have been conventionally classified into psychrophiles (cold loving), psychrotrophs (cold tolerant), mesophiles (moderate temperature loving), and thermophiles (heat loving) (Tortora et al., 2010). Psychrophiles are broadly distributed and have developed successful strategies to colonize cold environments and even regions under temperature fluctuations (De Maayer et al., 2014). These strategies include the upward regulation of some genes, enhanced membrane transport, increased biosynthesis of carotenoid pigments, and compatible osmotic solutes (De Maayer et al., 2014; Cray et al., 2015).

Nevertheless, the main actors in these adaptive responses are the proteins, which have a key role in the molecular mechanisms of the cell. They control the balance between nutrients and products, the assembly of macromolecules, the dynamic of nucleic acids, and the correct folding of other proteins (D’Amico et al., 2006). Thus, changes in the composition of the proteome in response to cellular stress conditions are linked with specific adaptive strategies (Maier et al., 2011). Chaperones are a group of essential proteins involved in the quality control of cell machinery (Hartl and Hayer-Hartl, 2002) and in dealing with the consequences of stress (Cooper and Hausman, 2000; Hochachka and Somero, 2002; Beckerman, 2005; Cloutier and Coulombe, 2013). They are constituent proteins responsible for the correct folding and building of other proteins and for eliminating those incorrectly formed during synthesis or as a result of a cell stress situation (Hartl and Hayer-Hartl, 2002; Camberg et al., 2013).

Among the chaperone family, heat-shock proteins (Hsp) play a well-known role in the prevention of aberrant protein aggregates as well as in the facilitation of protein synthesis, translocation, *de novo* folding, and the assembly of multi-protein complexes (Bromfield and Nixon, 2013). Their amount increases in the cell during stress situations like changes in the temperature of the environment. Not only does heat induce these proteins but the exposure of cells to low temperature can also lead to the synthesis of some Hsps (Hochachka and Somero, 2002; García-Descalzo et al., 2011). The main families of Hsps are highly conserved and classified based on their sequence and their molecular weight (Makhnevych and Houry, 2013). Two large and important families are Hsp70 and Hsp60 (Cooper and Hausman, 2000; Beckerman, 2005). Sometimes, members of these families act together sequentially during protein transport and folding of newly synthesized proteins (Langer et al., 1992; Cooper and Hausman, 2000). Protein DnaK is the main representative of the Hsp70 family in the prokaryotes (Thompson et al., 2012), and it is part of a chaperone system together with other co-chaperones, such as DnaJ, from the Hsp 40 family, and the nucleotide exchange factor GrpE (Ahmad et al., 2011; Perrody et al., 2012; Thompson et al., 2012). In the Hsp60 family, a great number of chaperone complexes are activated by cellular stress, including the GroEL-GroES complex (Ventura et al., 2004), which are responsible for facilitating the folding of other proteins from an incorrect or unfolded conformation to their native form in stressful situations (Cooper and Hausman, 2000).

Extremophiles employ different adaptive approaches to cope with environmental changes (Lin, 2008; Metpally and Reddy, 2009; Paredes et al., 2011). Their comparison is a very valuable tool to identify crucial features of adaptation to cold and fluctuating environments. With this purpose, we designed a comparative proteomic study of three bacteria: a mesophile, a psychrophile, and a psychrotroph. They were subjected to different growth temperature conditions to study the underlying molecular actors that are activated under diverse environmental scenarios. Two of these bacteria belong to the same genus but live in environments with different temperatures, while the other two share the same environment but belong to a different genus. In this way, our findings will clarify to what extent the strategies used by these microorganisms are derived from a similar genome or a similar environment. Detailed information about each strain is summarized in Table 1.

**TABLE 1:** Main features of the species used.

Specie	Taxonomy	Thermal classification	Optimal growth T^a^	Environment isolation	Available sequenced genome	Total proteome size
*S. oneidensis*	γ-Proteobacteria	mesophile	30°C	Oneida Lake (New York)	Yes (4,97 Mbp)	4196
*S. frigidimarina*	γ-Proteobacteria	psychrotroph	20–22°C	Antarctic sea ice	Yes (4,85 Mbp)	4030
*P. frigidicola*	γ-Proteobacteria	psychrotolerant	15°C	Antarctic ornithogenic soil	No (2,85Mbp)	2298

Moreover, the knowledge of these molecular mechanisms will have application in different research fields, including biotechnology, medicine, astrobiology, ecology, and climate change investigations (Cavicchioli et al., 2002). The molecules involved in these mechanisms range from enzymes that remain active with high efficiency at very low temperature to be used in the food industry (or in cosmetic or textile detergent production) (Cavicchioli et al., 2002) to biosensors that can report about the state of an ecosystem (Irwin and Baird, 2004), providing valuable information related to climate change or even to biomarkers of habitability in planetary exploration and planetary analog research.

## Materials and Methods

### Strains and Culture Conditions

Bacteria included in this study were purchased from culture collections: *Shewanella oneidensis* (MR-1, 700550TM) and *Shewanella frigidimarina* (ACAM 591, 700753TM) from the Germany Microorganisms and Culture Cell collection (DMSZ) and *Psychrobacter frigidicola* (ACAM 304, 700361TM) from the American collection (ATCC^®^). The two first strains belong to the same genus but are distinct regarding their optimal temperature condition and natural environment. *S. oneidensis* is a mesophile, while *S. frigidimarina* is a psychrotolerant and *P. frigidicola* belongs to a different genus and is a psychrophile.

Individual pre-inocula of the three strains were prepared from vials stored in glycerol at −80°C. Aliquots of 100 μl from these vials were incubated at their corresponding optimal growth temperature in tubes with 5 ml of each specific broth (BactoMarine Broth Difco2216 – MB – for *S. frigidimarina* and *P. frigidicola* and Luria-Bertani Broth – LB – for *S. oneidensis*). Once they reached the half exponential phase (corresponding to an optical density (O.D.) at 600 nm about 0.5), 500 μl of each one was inoculated in 250 ml Erlenmeyer flasks with broth at a final volume of 100 ml. Flasks of each species were incubated in triplicate at each experimental temperature of 4, 12, and 30°C for 5 days in aerobic conditions (150 rpm stirring). The O.D. at 600 nm was checked two times per day until it reached the stationary growth phase. Then, pellets of each flask were obtained by centrifugation for 20 min (10,000 × *g*; 4°C) and washed with phosphate buffer saline (PBS). Pellets were used to extract proteins for subsequent experiments. For the growth curves, statistical analyses were performed using GraphPad Prism version 7.0 (GraphPad Software, La Jolla, CA, United States^1^). The data were the mean ± SD from three replicates.

### Sodium Dodecyl Sulfate-Polyacrylamide Gel Electrophoresis and Western-Blot

Proteins from the pellets were extracted to study the potential differences in protein profiles of the three bacteria against different temperature conditions. The preparation of cell extracts and protein determination were performed as described in García-Descalzo et al. (2014).

Protein extracts were separated and analyzed by sodium dodecyl sulfate-polyacrylamide gel electrophoresis (SDS-PAGE) for 1 h at 200 V and 400 mA (PowerPac 200, BioRad) in 12% acrylamide-bisacrylamide gels (2.6% crosslinking) using low molecular weight (LMW) (GE Healthcare) marker. Gels were stained with Coomassie Blue or transferred to polyvinyl-difluoride membranes (PVDF) to perform chaperones immunodetection by western blot (WB).

Membranes were firstly incubated overnight in a cold chamber with antibodies anti-DnaK, anti-GroEL, anti-DnaJ, and anti-GroES (Table 2). A second incubation was performed with HRP-linked anti-rabbit or anti-mouse (1:1,000) for 1 h. After several washes with PBS, membranes were revealed by chemiluminescence using ECL Western blotting detection reagents (GE Healthcare).

**TABLE 2:** Antibodies for WB and the dilution used.

Chaperone antibody	Protein MW	Host	Type	Dilution
DnaK	70 kDa	mouse	monoclonal	1:1000
GroEL	60 kDa	mouse	monoclonal	1:1000
DnaJ	41 kDa	rabbit	policlonal	1:5000
GroES	10 kDa	rabbit	policlonal	1:10000

### Two-Dimensional Fluorescence Difference Gel Electrophoresis

The proteins produced in each bacterium at the analyzed temperatures were separated by 2-DE (two-dimensional electrophoresis). Samples (150 μg from each extract) were prepared as previously described (García-Descalzo et al., 2014) with slight modifications. After the first dimension by IEF (isoelectric focusing), which separates proteins based on their isoelectric points, IPG-strips (Immobiline Dry Strips, GE; pH 3-11 NL – non-linear – of 24 cm) were equilibrated in two successive steps with SDS equilibration buffer solutions containing a Tris buffer, 100 mM; urea, 6 M; glycerol, 30% (w/v); and DTT, 0.5% (w/v). Then, they were incubated with iodoacetamide, 4.5% (w/v). The second dimension in SDS-PAGE, to separate proteins by their molecular weight, was carried out on 12% acrylamide (2.6% crosslinking) gels (1.-mm thick). Gels were stained with Coomassie Blue or with MALDI-MS-compatible silver reagent for protein identification (Miller et al., 2006).

To compare the proteins over and under synthesized in each temperature condition, proteins from bacterial cells grown at 4 and 30°C were analyzed by 2-D DIGE. Proteins were extracted from the cultures as indicated before, obtaining four biological replicates of each one. Samples (40 μg) from each experimental condition were made using 7 M urea/2 M thiourea and labeled with DIGE Fluor minimal dyes (GE Healthcare) for their electrophoresis separation and analysis. Two replicates of each experimental condition were labeled with Cy3 and the other two with Cy5. A pool of both temperature conditions was also labeled with Cy2 to be used as an internal standard.

Protein gel images were processed and analyzed using the DeCyder Differential In-gel Analysis (DIA). The spots were co-detected, quantified, normalized, and matched between the four replicate experiments. Protein abundance changes between samples from each strain grouped by the temperatures 4 and 30°C (4/30 ratio) were examined by ANOVA test. Spots present in at least three of four gels per group, with significant ANOVA test (*p* ≤ 0.05 or *p* ≤ 0.01), and an averaged 4/30 ratio ≥ ± 2 were considered and selected for further MS analysis in preparative gels. Those spots selected in new gels were excised, digested with trypsin, and identified by matrix-assisted laser desorption/ionization time-of-flight mass spectrometry (MALDI-TOF MS).

The identification protocol and data search used were based on the method previously described in Cid et al. (2010) and García-Descalzo et al. (2014) with minor variations. Combined peptide mass fingerprint and MS/MS ion search modes were applied against the NCBInr databases (NCBInr 081217 with 7463447 sequences and 2570742364 residues for *S. oneidensis*; NCBInr 160909 with 9694989 sequences and 3312496757 residues for *S. frigidimarina*; NCBInr 20131020 with 33055681 sequences and 11532217697 residues and NCBInr 14042010 with 10866589 sequences and 3703552722 residues for *P. frigidicola*). The MASCOT^MT^ (Matrix Science, London, United Kingdom) database search algorithm was used for protein identification (search parameters: Enzyme-trypsin; fixed modifications carbamidomethyl (C); variable modifications-oxidation (M); mass values-monoisotopic; protein mass unrestricted; peptide mass tolerance-±50 ppm; peptide charge State- 1+; max-missed cleavages-1).

### Immunoprecipitation

Cell extracts were immunoprecipitated with monoclonal anti-DnaK and anti-GroEL antibodies (Enzo Life Sciences, United States) to study the DnaK- and the GroEL-associated proteins in the three bacteria studied.

The immunoprecipitation was carried out using Protein G-Sepharose previously washed with PBS. In the first-step, 600 μg of protein extract from each sample was mixed with 3 μl (1 mg/ml) of an antibody (anti-DnaK or anti-GroEL) for 4 h at 4°C with rotation in an immunoprecipitation buffer (IPB) (Tris HCl, 500 mM; pH: 7.5; NaCl, 200 mM; 0.2% Triton X-100; 0.2% NP-40; EDTA, 5 mM; pH: 8, protease inhibitors – Roche – and phenylmethylsulphonyl fluoride, PMSF, 1 mM). The immunoprecipitation was developed in stringent conditions to minimize unspecific complexes. Second, 30 μl of washed Protein-G-Sepharose (50% of slurry) was added, followed by 1 h of incubation in the same previous conditions. Finally, the immunoprecipitates were washed two times in a cold isotonic protease inhibition buffer (IPIB) (Tris HCl, 1 M; pH: 7.5; EDTA, 0.2 M; NaCl, 150 mM; 0.2% SDS) with protease inhibitors (Roche) to remove the unspecific binding. Then, samples were centrifuged for 5 min at 5,000 rpm, discarding the supernatant. The solid phase was subjected to two-dimensional electrophoresis (2 DE), and the subsequent protein identification was carried out by MALDI-TOF MS or kept at −20°C until use.

## Results

### Cell Growth at 4 and 30°C (and 12°C)

**FIGURE 1:**
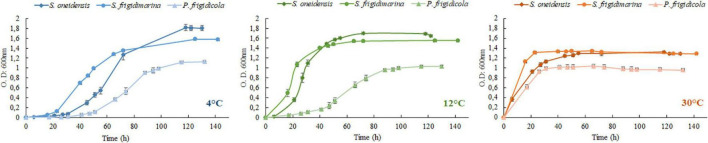
Growth curves of *S. oneidensis*, *S. frigidimarina*, and *P. frigidicola* at 4 (blue), 12 (green), and 30°C (red/orange).

Despite having different optimal temperatures for growth, the three bacteria studied were able to adapt to changing laboratory conditions, mimicking some of the extreme temperatures they could experience in their natural ecosystems. Their growth achieved high O.D. values in the established temperature range, although their lag phase was very different (Figure 1 and Table 3). In the three bacteria, the growth at 4°C demanded a large lag phase; while warmer temperatures depicted a quicker onset, regardless of whether they were mesophile, psychrotolerant, or strict psychrophile. The differences between lag phase duration were statistically significant for the three strains at 4°C as well as for the case of *P. frigidicola* compared to the other two strains. The exponential phase duration when bacteria grew at 4°C was considerably larger when they grew at 30°C for the three species. Growth rates of each strain at each temperature treatment showed significant differences between *S. frigidimarina* and *S. oneidensis*, growing at 4 and 30°C and between *S. frigidimarina* and *P. frigidicola*, growing at 12 and 30°C (Table 4). For the two Shewanellas, the highest rate appeared at 30°C, while the rate of *P. frigidicola* is quite similar in the three temperatures tested.

**TABLE 3:** Duration of the lag phase in each bacterium at each temperature studied.

		4°C	12°C	30°C
*S. oneidensis*		32.00 (0.10)	7.00 (0.10)	0
*S. frigidimarina*		23.00 (0.10)	4.00 (0.11)	0
*P. frigidicola*		47.33 (0.33)	44.67 (3.22)	0
*r* ^2^		0.9993***	0.9993***	NS
Multiplecomparison	*S. oneidensis* vs. *S. frigidimarina*	***	NS	NS
	*S. oneidensis* vs. *P. frigidicola*	***	***	NS
	*S. frigidimarina* vs. *P. frigidicola*	***	***	NS

Data are expressed as mean ± (SD). Statistical differences were studied by ANOVA test (***p ≤ 0.0001). Statistical significance was achieved by Bonferroni’s post-test (NS, not significant;***p ≤ 0.001).

**TABLE 4:** Growth rates (h^–1^) of *S. oneidensis*, *S. frigidimarina*, and *P. frigidicola* at 4, 12, and 30°C.

		4°C	12°C	30°C
*S. oneidensis*		13.91 (0.71)	8.777 (1.91)	11.37 (1.07)
*S. frigidimarina*		19.86 (1.22)	6.29 (2.15)	32.19 (2.28)
*P. frigidicola*		16.73 (1.47)	12.12 (2.15)	16.03 (4.42)
*r* ^2^		0.8646**	0.7370*	0.9328***
Multiplecomparison	*S. oneidensis* vs. *S. frigidimarina*	**	NS	***
	*S. oneidensis* vs. *P. frigidicola*	NS	NS	NS
	*S. frigidimarina* vs. *P. frigidicola*	NS	*	**

Data are expressed as mean ± (SD). Statistical differences were studied by ANOVA test (*p ≤ 0.01; **p ≤ 0.001; ***p ≤ 0.0001). Statistical significance was achieved by Bonferroni’s post-test (NS, not significant; *p ≤ 0.05; **p ≤ 0.01; ***p ≤ 0.001).

### Synthesis Levels of Chaperones With Temperature Changes Immunodetected by Western Blot

**FIGURE 2:**
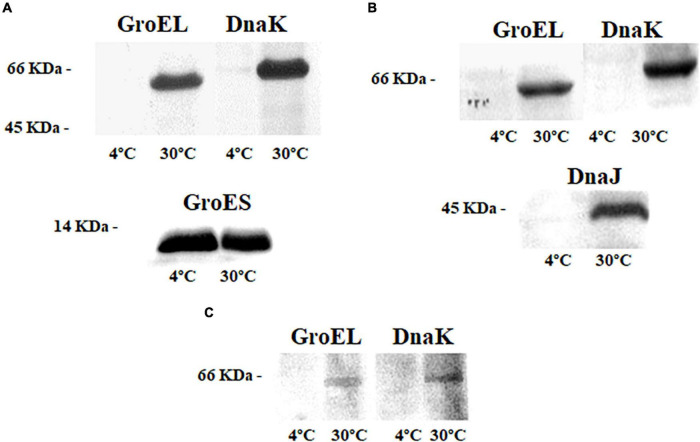
Immunodetection of chaperones in the cell extracts of the three bacteria at 4 and 30°C; **(A)** GroEL, DnaK, and GroES in *Shewanella oneidensis*; **(B)** GroEL, DnaK, and DnaJ in *Shewanella frigidimarina*; and **(C)** GroEL and DnaK *Psychrobacter frigidicola*.

The relative synthesis levels of chaperones GroEL, GroES, DnaK, and DnaJ at 4 and 30°C for each bacterium were studied by WB. Cultures of the mesophile, *S. oneidensis*, at 30°C showed strong signals of chaperones GroEL/GroES and DnaK (Figure 2A). Cultures at 4°C revealed a weak band corresponding to DnaK, while a strong immunodetection of chaperonine GoES was observed (Figure 2A). These results may suggest that the machinery activated and controlled by these chaperones could be an advantage for the mesophile bacterium, allowing it to thrive in cold conditions and to grow even at temperatures far from its optimum.

The chaperones GroEL, DnaK, and DnaJ were immunodetected in samples from *S. frigidimarina* cultured at 30°C (10°C above their optimal growth temperature). Nevertheless, the immunodetection of chaperones in cultures at 4°C was not possible (Figure 2B).

In this case, the chaperone system at 30°C was formed by the chaperone DnaK from the Hsp70 family and its co-chaperonin DnaJ from the Hsp40 family. No levels of the co-chaperonin GroES were detected, despite the strong detection of its partner GroEL.

The immunodetection in samples from cultures of the psychrophile *P. frigidicola* showed detectable levels of GroEL and DnaK chaperones when cells were grown at 30°C but not at 4°C (Figure 2C). The optimal growth temperature for this microorganism is 15°C. It seems that it needs these chaperones to cope with warm temperatures, while, at cold temperatures, the levels of these proteins remained undetectable by WB.

### Identification of Over- and Under-Synthesized Proteins at Warm and Cold Temperatures

*Two-dimensional fluorescence difference gel electrophoresis* experiments were performed to compare the whole proteomes of each bacterial species cultured at 4 and 30°C. In that way, the molecular machinery used in the adaptation to changes in temperature could be studied.

#### Two-Dimensional Differential Electrophoresis Analysis of *Shewanella oneidensis* at 4 and 30°C

2DE-labeled images (Supplementary Figure 2) were matched to analyze the results of 2D-DIGE as explained above. The fluorescence intensity of 395 protein spots was significantly altered by an averaged fold change of ±2 (*t*-test, *p* < 0.01). The 51 spots, which showed the greatest change, were selected and excised from the gel (Supplementary Figure 3), digested with trypsin, and identified by MALDI-TOF or MALDI-TOF-TOF MS. Finally, from the 51 spots selected, 44 were identified corresponding to 47 proteins, while 7 spots could not be identified (Table 5).

**TABLE 5:**
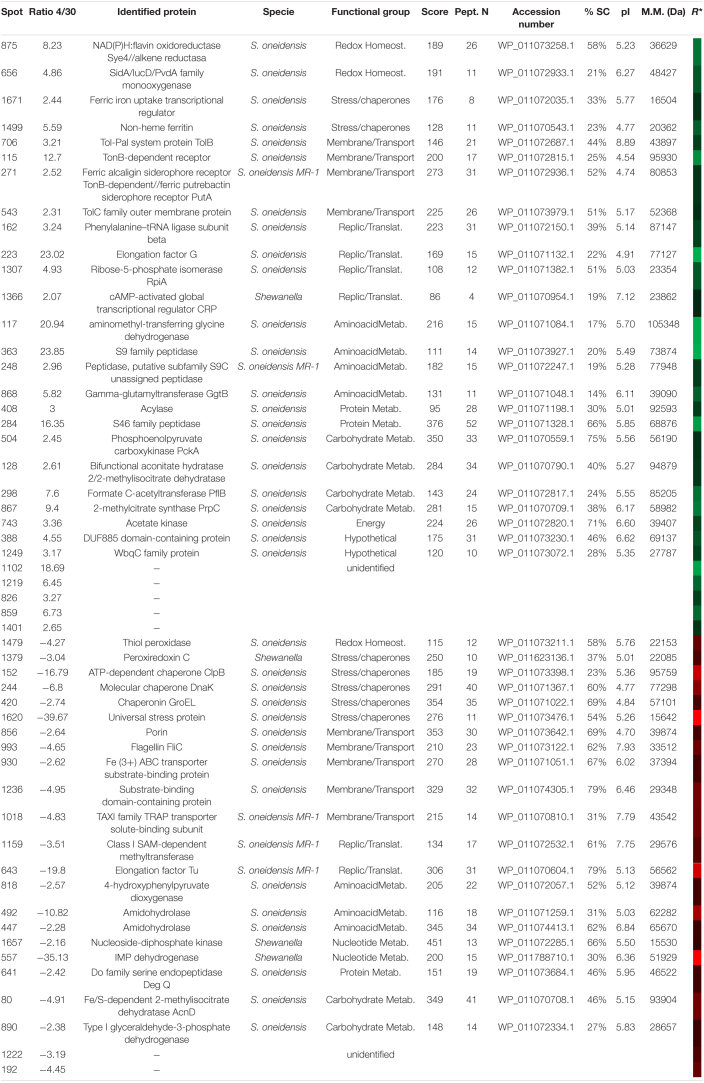
Identified proteins in *S. oneidensis* overexpressed at 4 (green) and 30°C (red).

**FIGURE 3:**
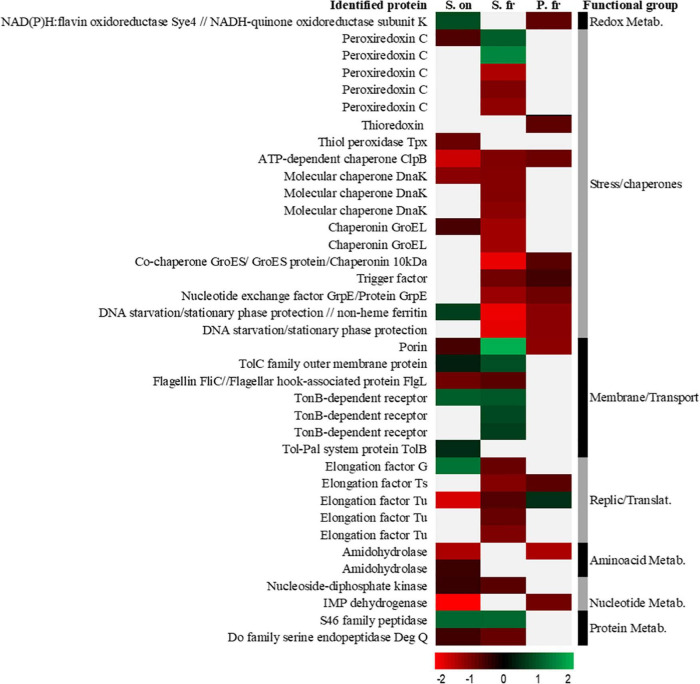
A heat map of ratios from 2D-DIGE experiments of the proteins over-synthesized at 30 (red) and 4°C (green) found in common in at least two of the three studied bacteria. The color and intensity of each protein are calculated as a function of the logarithm based on 10 of the value of its 4/30 ratio obtained in the 2D-DIGE experiment so that values closer to –2 indicate higher ratios in proteins whose synthesis has been increased to 30°C and closer to 2 higher ratios in proteins whose synthesis has been increased to 4°C. The gray/white color indicates the absence of identification of that specific protein in the corresponding species. “*S. on*” indicates *Shewanella oneidensis*, “*S. fr*” indicates *Shewanella frigidimarina* and “*P. fr*” indicates *Psychrobacter frigidicola*.

R* represents a heatmap of the ratio 4/30 as in Figure 3. A specie column refers to the organisms (specie or genus) in which protein has been identified according to the NCBI database.

The identified proteins were classified into functional groups: carbohydrate metabolism, amino acid metabolism, nucleotide metabolism, protein metabolism, energy, redox homeostasis, transport and transmembrane proteins, chaperones and stress response, replication and translation, hypothetical, and an additional group one of the non-identified spots.

The main increased proteins observed at 30°C were especially from the categories related to stress response (GroEL, ClpB protein, DnaK), transport, and membrane proteins [flagellin, iron (III) ABC transporter, substrate-binding domain-containing protein], TAXI family TRAP transporter solute-binding subunit, and outer membrane porine. In cultures, at 4°C, *S. oneidensis* increased the synthesis of groups involved in replication and translation processes and in the biosynthesis of proteins and aminoacids precursors (Phe-tRNA, elongation factor G (EF-G), RpiA, and the cAMP-CRP). These bacteria also showed an increase in redox enzymes involved in the response to oxidative stress like Flav-Sye4.

#### Two-Dimensional Differential Electrophoresis Analysis of *Shewanella frigidimarina* at 4 and 30°C

After matching the fluorescence images (Supplementary Figure 4) and normalizing data from conditions of 4 and 30°C, 128 protein spots showed significant differences based on changes in the abundance ratio of ±1.5-fold with a *t*-test of *p* < 0.005. The 43 with the greatest differences were selected and cut off for their identification. About 42 spots were identified (Supplementary Figure 5) corresponding to 31 proteins – some of them with several isoforms – and 5 without satisfactory identification (Table 6).

**TABLE 6:**
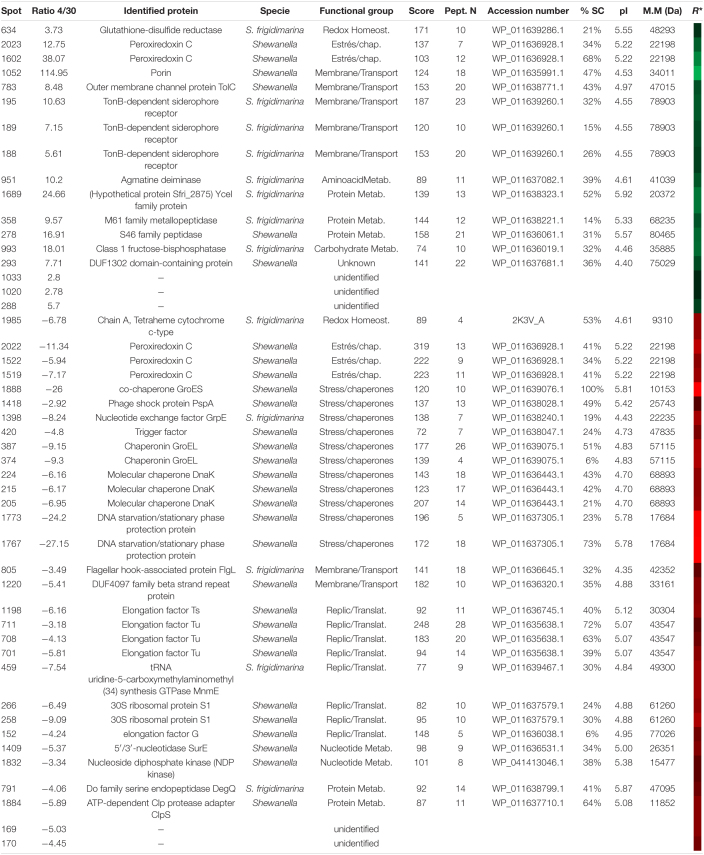
Identified proteins in *S. frigidimarina* overexpressed at 4 (green) and 30°C (red).

R* represents a heatmap of the ratio 4/30 as in Figure 3. A specie column refers to the organisms (specie or genus) in which protein has been identified according to the NCBI database.

Samples at 30°C showed a greater number of proteins identified as over-synthesized. The functional groups more represented were chaperones and stress proteins (GroES, Trigger factor, PspA, Clps adaptor, GrpE, GroEL, and DnaK) as well as the replication and translation proteins (elongation factors and ribosomal proteins). However, these two groups were not over-synthesized in samples at 4°C. At this temperature, the main groups identified were those of transport and membrane proteins. It is noteworthy that, in the 30°C samples, the redox metabolism was represented mainly by the appearance of three isoforms of the peroxiredoxin C protein. Two other isoforms of the same protein also appeared in cultures at 4°C.

Unlike the other two tested bacteria, cultures of *S. frigidimarina* at both 4°C and 30°C show a high number of isoforms of different proteins [TonB-dependent receptor, peroxiredoxin C, GroEL, DnaK, ferritin, elongation factor Tu (EF-Tu) and RpS1].

#### Two-Dimensional Differential Electrophoresis Analysis of *Psychrobacter frigidicola* at 4 and 30°C

The merged fluorescence images were analyzed (Supplementary Figure 6) and normalized. The data from the extracts at 4 and 30°C determined the protein spots of greatest interest. Thus, based on changes in the abundance ratio of ±2.5 times fold between both temperature conditions and with a *p* < 0.05 as statistically significant, 86 spots were selected. From them, the 39 spots with the greatest differences were cut out (Supplementary Figure 7), resulting in 36 (Table 7) proteins identified and 5 with no satisfactory identification. Due to the recent sequence project of this species, the identification of spots was carried out using databases of the closest phylogenetic species, such as *Psychrobacter cryohalolentis*, *Psychrobacter arcticus*, or the genus *Psychrobacte*r in other cases.

**TABLE 7:**
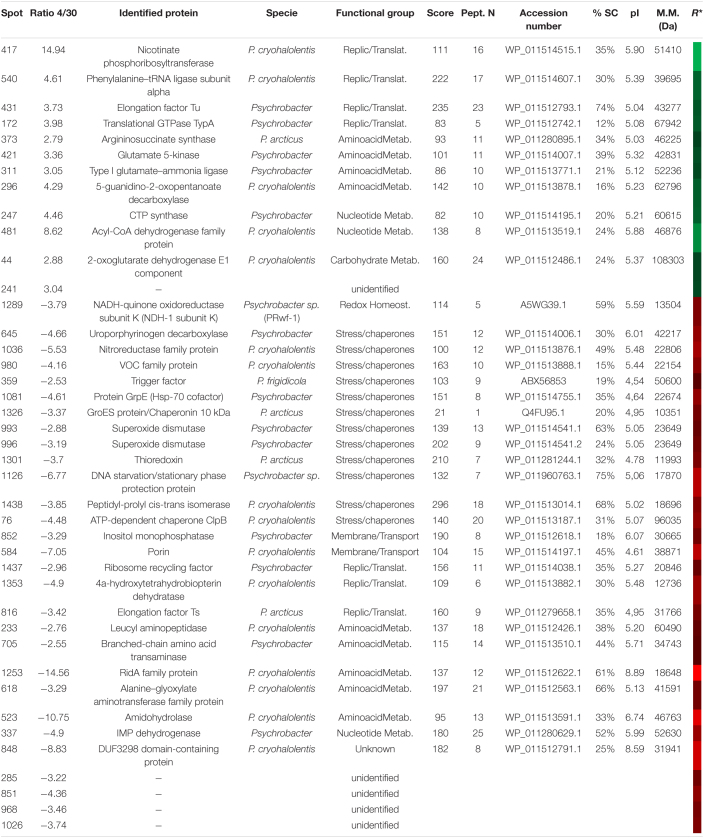
Identified proteins in *P. frigidicola* overexpressed at 4 (green) and 30°C (red).

R* represents a heatmap of the ratio 4/30 so the level of overexpression in each case being more brilliant at higher values and darker at lower. A specie column refers to the organisms (specie or genus) in which protein has been identified according to the NCBI database.

In this species, we identified more than double the number of proteins over-synthesized at 30°C with regard to 4°C. Similarly to *S. frigidimarina*, the main group represented at 30°C, that was not found at 4°C, corresponded to *chaperones and stress proteins* [trigger factor, GrpE, GroES, superoxide dismutase (SOD), thioredoxin, ferritin, peptidyl-prolyl isomerase, and ATPase AAA-2]. Besides, we found, at 30°C, several proteins were involved in redox metabolism that did not appear in the 4°C condition. The main groups over-synthesized in cultures at 4°C were those of transport and transmembrane proteins, like TolC (spot 783), three isoforms of TonB (188, 189, and 195), and porin (1052).

#### Proteins in Common Revealed by Two-Dimensional Fluorescence Difference Gel Electrophoresis

Those proteins identified in at least two of the three species were grouped by a functional category, comparing their synthesis through changes in the 4/30 ratio and represented in a heatmap (Figure 3).

The protein groups most shared between the three bacterial species were those related to thermal stress, oxidative stress, and transmembrane transport. Mainly, the heatmap showed more coincidences in the production of proteins regarding temperature between *S. frigidimarina* and the other two species than between the other two species.

### Comparison of Molecular Machinery

**FIGURE 4:**
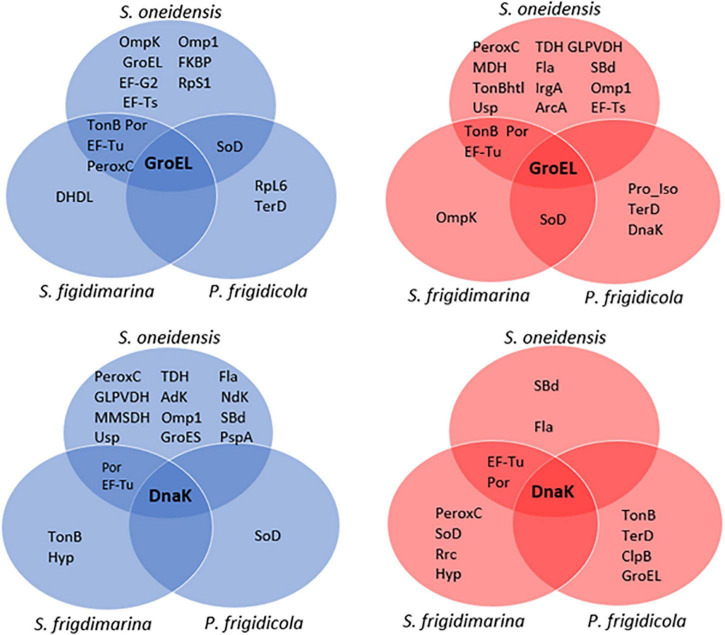
Venn diagrams of identified proteins that coimmunoprecipitate with GroEL (top) and DnaK (down) in the three bacteria at 4 (blue) and 30°C (red).

To compare the proteins *in vivo* linked to GroEL and DnaK chaperones, immunoprecipitations were carried out with specific anti-GroEL and anti-DnaK antibodies. The immunoprecipitates were analyzed by 2DE, and proteins were identified by mass spectrometry (MS). The extended list of proteins identified that co-immunoprecipitate with DnaK or GroEL in each species and at each temperature are summarized in Supplementary Material (Supplementary Tables 1–4). Figure 4 represents a comparison of the molecular machinery expressed by each bacterial species at each temperature tested that interacts with chaperones GroEL or DnaK.

Regarding the proteomic machinery, *P. frigidicola* and *S. oneidensis* used different proteins in these interactions. At warm temperatures, they only had one common interaction (GroEL with SOD). On the other hand, the machinery with more proteins in common was found between the two shewanellas at both temperatures and was related to both DnaK and GroEL. These proteins were elongation factors and transmembrane proteins, and the peroxiredoxin C in the case of immunoprecipitation with GroEL in cultures at 4°C. Specifically, *S. oneidensis* displayed a higher number of proteins interacting with chaperone GroEL, at 4 and 30°C, and DnaK, at 4°C, than *S. frigidimarina* and *P. frigidicola*. In contrast, the immunoprecipitation with DnaK in samples at 30°C showed a slight increase in the number of proteins identified in the species *S. frigidimarina* and *P. frigidicola*, compared to *S. oneidensis*. In fact, *P. frigidicola* immuno precipitated more proteins at 30°C with antibodies from both chaperones used than at 4°C, while *S. frigidimarina* showed a similar number of proteins interacting with both chaperone antibodies at 4 and 30°C, with the exception of a slight increase in the case of DnaK at 30°C.

## Discussion

The aim of this study was to understand the molecular strategies of three bacteria to cope with temperature changes in their environment. Given that the proteins are the main actors in these strategies and are responsible for the active machinery of the cells (D’Amico et al., 2006), the investigation has mainly focused on them. As mentioned above, this was achieved by comparing the behavior of psychrotolerant bacteria (i) *Shewanella frigidimarina*, which share a great part of the genome with the mesophile (ii) *Shewanella oneidensis* and came from the same environment (Antarctica) as the third bacteria (iii) *Psychrobacter frigidicola* from a different genus.

### Growth Dynamics Under Different Temperatures

On the whole, the behavior of the three bacteria studied regarding their growth and biomass multiplication under cold, mild, and warm temperatures appeared relatively similar, with only mild differences in their growth phases (Table 3) and growth rates (Table 4). These differences could inform whether the bacteria are in a normal or stressful situation, taking into account the length of the lag phase. However, this would be just a potential finding taken as sole data (Hamill et al., 2020) that would need to be supported with further analysis by proteomic experiments.

*Shewanella oneidensis* is considered a mesophile according to its optimal growth temperature (Venkateswaran et al., 1999), but it was originally isolated from Oneida Lake in New York, which is frozen or at temperatures near 0°C for almost half of the year. This could be the reason why it can multiply at 4°C after a long lag phase with a relatively similar growth rate than at 30°C. Besides, this bacterium can behave as a psychrotolerant after a preadaptation period (Hébraud and Potier, 1999) as it has also been described for *E. coli* (Jones et al., 1987) and other mesophiles isolated from the Arctic and Antarctic permafrost (Steven et al., 2006).

*S. frigidimarina* and *P. frigidicola*, whose preinocula were set at 20°C, and *S. oneidensis* (preinocula at 30°C) showed marked lag phases in their growth at 4 and 12°C. In general, cold-adapted bacteria possess regulating factors that keep the translating machinery active at low temperatures. When their growth pauses due to a shift in temperature, they can activate these regulating mechanisms after an adaptation period and grow again at these new low temperatures (Hébraud and Potier, 1999). For *S. frigidimarina*, this accommodation period is shorter at 12°C than at 4°C, probably due to the small temperature difference from its optimal growth temperature, although the growth rates are different, being higher at 4°C. This may indicate that the strategies activated during the lag period provide efficient molecular machinery for successful bacterial development. The ACAM 304 strain of the psychrophile *P. frigidicola*, which has an optimal growth temperature of 15°C, was isolated from ornithogenic soil in Antarctica (Bowman et al., 1996). Its behavior at 4 and 12°C, with relatively long lag phases, could indicate that it has a generalized decrease in affinity at low temperatures (Nedwell and Rutter, 1994). This bacterium is described as a strict psychrophile from an environment where it is competing with psychrotolerants, which are predominant in polar areas (Nedwell and Rutter, 1994) and are better adapted to temperature changes and fluctuations (Baross and Morita, 1978).

In this study, we have observed that the growth of the three species at warm temperatures (30°C), with almost no lag period, implies a fast entry in the stationary phase, especially for the psychro-species. Nevertheless, and contrary to what one might expect, the psychrotolerant and psychrophilic bacteria showed an effective growth at temperatures slightly higher than those of their described limits. The reason could be the well-known effect of the adaptation of laboratory strains. This can expand cellular limits of medium/environmental conditions (Knöppel et al., 2018), susceptibility against chemicals (Herruzo-Cabrera et al., 2004), or even temperature, such as *Saccharomyces cerevisiae*, which can increase their maximum growth temperature by 3°C (Caspeta and Nielsen, 2015). Furthermore, the growth of the three bacteria at low temperatures reached higher values of O.D., probably due to the improved efficiency in the use of substrates during the first period of incubation, which allows psychrophiles to reach maximum amounts of biomass (Morita, 1975). Microbial growth is the result of a sequence of interrelated chemical reactions (Hébraud and Potier, 1999), and is not only influenced by temperature but also by the availability and quantity of nutrients (Russell, 1990), especially important in the late exponential phase and the entry into the stationary phase.

### Changes in Synthesis Levels of Chaperone Systems GroEL/GroES and DnaK/DnaJ With the Temperature

Chaperones GroEL and DnaK have a crucial role in the adaptation to thermal stress through refolding of denatured or misfolded proteins, avoiding the formation of damaging aggregates, or eliminating them by proteolysis (Gragerov et al., 1992; Phadtare, 2004; Robin et al., 2009). On many occasions, they act together in successive steps being the pair DnaK/DnaJ, which first recognizes the substrate protein and stabilizes an intermediate conformation. Subsequently, the pair GroEL/GroES acts by ATP hydrolysis to conform to the native state of the protein (Langer et al., 1992).

The immunodetection in samples at 30°C showed high levels of different chaperones in each strain (GroEL/GroES and DnaK in *S. oneidensis*, GroEL, DnaK/DnaJ in *S. frigidimarina* and GroEL and DnaK in *P. frigidicola*), suggesting that this warm temperature condition represents a stressful situation that promotes the increase of Hsps levels (Lüders et al., 2009; Che et al., 2013). As indicated before, the natural environment of *S. oneidensis* is a lake that remains frozen or at temperatures near 0°C for 6 months of the year. This bacterium, although described as a mesophile, is able to behave as a psychrotolerant, showing similarities with *S. frigidimarina* in responses to temperature conditions. It is also known that mesophiles and psychrotolerants sometimes overlap with their thermal properties (Russell, 1990). The signal of GroES immunodetected in *S. oneidensis* growing at 4°C was relatively strong, which correlates with the fact that this chaperone is greatly produced at low temperatures in some species of the *Shewanella* genus (Miyake et al., 2007), although, in *S. frigidimarina*, this chaperone was not detected. *S. frigidimarina* and *P. frigidicola*, which are both originally from Antarctica, had undetectable levels of the chaperones tested in cultures at 4°C, suggesting that, even far from their respective optimal growth temperatures (20°C for *S. frigidimarina* and 15°C for *P. frigidicola*), they do not require the activity of these Hsps to cope with a cold environment such as at 4°C.

### Over-Synthesis of Proteins at 4 and 30°C in the Three Bacteria

#### Over-Synthesized Proteins at Low Temperature

Cultures of the psycrotrophic *S. frigidimarina* at 4°C produce mainly a group of proteins related to the low iron availability or its metabolism, membrane transport, and oxidative stress. At low temperatures, the solubility of gases, especially oxygen, increases, and free radicals are more stable. Psychrophiles possess several mechanisms to cope with the oxidative stress of these reactive oxygen species (ROS). These mechanisms include the synthesis of specific reductases to repair oxidized residues, reduction in the number of oxidizable amino acids in the proteins, and the deletion of metabolic pathways that produce ROS (Piette et al., 2010). The increase of proteins like peroxiredoxin C and GDHr, which are involved in the redox homeostasis of the cell by acting as a reducing disulfide (Aslund and Beckwith, 1999), is presumably triggered to restore reduced cell conditions occurring at low temperatures. Membrane proteins identified in these cultures, system TolC-TonB, conform a membranal system to bond, transport, and chelate iron into the cell (Krewulak and Vogel, 2011), thus regulating iron homeostasis. Since cold affects the membrane fluidity (Bajerski et al., 2017; Siliakus et al., 2017), it could also be prompting these proteins for a better regulation of iron homeostasis to be used as a cofactor for the function of other proteins and respiration and to avoid more ROS emergence (Yang et al., 2008).

Similar to *S. frigidimarina*, when *S. oneidensis* grew at low temperature, there was an increase in the synthesis of redox enzymes and iron metabolism-related, as well, to an increase in proteins of the transport system, consisting of Ton-B-dependent receptor protein (Spot 115), the Tol-Pal system protein TolB (Spot 706), and TolC (Spot 543). This may be an indicator of an oxidative stress situation at the cold that *S. oneidensis* and *S. frigidimarina* face due to the increase of proteins that regulate iron levels and its transport for cell respiration, and accurate homeostasis to avoid oxidative poisoning (Mirus et al., 2009).

Different groups of proteins involved in replication and translation processes and in the biosynthesis of other proteins and aminoacid precursors were also over-accumulated in *S. oneidensis* at 4°C. Among them, the one with a notably higher ratio (23.02) was EF-G, which plays a key role in the elongation of polypeptides during the synthesis of protein (Savelsbergh et al., 2009; Li et al., 2011). In *E. coli*, EF-G has been described as susceptible to oxidative stress (Nagano et al., 2012) and plays a chaperone role in protein folding (Caldas et al., 2000). Furthermore, its marked synthesis increase at 4°C in *S. oneidensis* could be because it behaves like a cold-adapted enzyme, with high molecular flexibility (Ruggiero et al., 2007) and a low activation energy requirement in GTP hydrolysis (Thomas and Cavicchioli, 2000), which is able to keep active at low temperatures.

At 4°C, this bacterium also increased the synthesis of proteins related to intracellular levels of acetyl-CoA. Bacteria often cope with changes in their environment by activating metabolic pathways to produce available nutrients. The phosphorylation of some regulating proteins in response to stress involves mechanisms that include acetyl phosphate, whose gene is induced by a shortage of phosphate in some bacteria (Summers et al., 1999). Temperature and the growth phase are factors that influence intracellular concentrations of acetyl phosphate (Prüss and Wolfe, 1994). Thus, the over-synthesis of enzymes that control intracellular levels of acetyl-CoA, like the acetate kinase, detected in *S. oneidensis* at 4°C, could respond to the need of the bacterium to generate energy and phosphorylate other proteins to be active in response to a stressful situation.

In this line, there was a remarkable increase in the production of the enzyme acyl-CoA dehydrogenase (ACDH) (Spot 481), also in *P. fridigicola* at 4°C that could support the activation of the metabolism of this bacterium at cold temperatures. An active metabolism contributes to the generation of energy and electron transfer in respiration with the participation of acetyl-CoA, a product of the reaction catalyzed by ACDH in metabolic pathways such as the tricarboxylic acid cycle. This enzyme belongs to the family of proteins involved in lipid oxidation and in the metabolism of amino acids and acts as an oxidoreductase for the transfer of electrons in the respiratory chain (Kim and Miura, 2004). Possibly, this active metabolism in *P. frigidicola* cultures at low temperature also requires a high synthesis and presence of NAD co-factors of the main metabolic processes (Gerdes et al., 2006). Interestingly, we did find strong induction of the enzyme nicotine-phosphoribosyl transferase (spot. 417) too, which catalyzes the first reaction in the synthesis of NAD (Dulyaninova et al., 2000).

The active metabolism of this bacterium at 4°C was also depicted by the over-synthesis of proteins involved in the biosynthesis processes of numerous amino acids and nucleotides, as well as in the metabolism of carbohydrates, while there was no induction of chaperons or stress proteins detected by 2D-DIGE. One of the most strongly induced proteins in these samples was the CTP synthase. This enzyme is responsible for the production of CTP from UTP and glutamine, and for the regulation of intracellular levels of CTP (Endrizzi et al., 2004), which is an essential precursor of membrane phospholipids (Chang and Carman, 2008). It also plays a key role in the metabolism of pyrimidines and is very important for the growth rate and the concentration of ribonucleotides and deoxyribonucleotides (Jørgensen et al., 2004).

#### Over-Synthesized Proteins at High Temperature

When *S. frigidimarina* grew at warm temperatures (30°C), an increase in the synthesis of several chaperones and stress response proteins was observed. They were mainly proteins that face oxidative, osmotic, and thermal stress situations. Some of them appeared in different isoforms like DnaK (posts 224, 215, 205) and GroEL (Spots 387 and 374) and others as single forms like GrpE (Spot 1398), TF (Spot 420), PspA (Spot 1418), and GroES (Spot 1888). The latter, although undetectable by WB in this study, forms the GroEL-GroES complex that keeps bound in the presence of ATP (Ventura et al., 2004) in the thermal stress response. These chaperones play an important role in the quality control system of post-translational processing. They control the folding and the proper conformation of other proteins to cope with environmental changes such as temperature, cooperating with each other (Liberek et al., 2008; Muga and Moro, 2008; Robin et al., 2009). In response to oxidative stress at warm temperatures, we found in *S. frigidimarina* two isoforms of the DNA-binding ferritin-like proteins region, DNA-Dps (spots 1773 and 1767), which belongs to Dps family proteins. This family is involved in protecting DNA from damage by chelating iron and by ferroxidase activity (Calhoun and Kwon, 2011). DNA-Dps is also required for starvation response and long-term stationary viability (Jeong et al., 2006) that is why we cannot discard some contribution to its high levels detected to the fact that the samples were collected once cultures reached the stationary phase. Nevertheless, another three isoforms of other protein-related to oxidative stress were also overexpressed in these samples, the peroxiredoxin C (Spots 2022, 1522, 1519). It is part of the large and highly conserved family of the peroxiredoxines, which detoxify peroxides by reducing them (Rhee, 2016). This protein is also known as alkyl hydroperoxide reductase (AhpC) described as a protective enzyme against peroxides in *E. coli* (Storz and Imlay, 1999) against osmotic stress in *Staphylococcus aureus* and induced by a temperature increase in *Bacillus subtilis*, while, in *Shewanella putrefaciens*, its activity is related to cold acclimation (Leblanc et al., 2003).

This molecular strategy involving proteins against redox, osmotic, and stationary phase stress during warm conditions seems to be common in the two shewanellas studied, being the peroxiredoxin C also identified as over-accumulated in warm cultures of *S. oneidensis* (Spot 1379). In this bacterium, at 30°C, there were too many other over-synthesized proteins related to oxidative and osmotic stress belonging to the anti-oxidant AhpC-TSA or peroxiredoxin family. That is the case of the Tpx-C (Spot 1479) that protects against oxidative (Storz and Imlay, 1999; Horst et al., 2010; Somprasong et al., 2012), osmotic and cold stress (Leblanc et al., 2003), and that increase in the late stationary phase (Miyake et al., 2007). Thus, the bacteria *S. oneidensis*, as well as *S. frigidimarina*, could share a similar mechanism to that of *Bacillus subtilis* in which this type of enzyme is induced as a response to temperature increase and osmotic stress after the beginning of the stationary phase (Hecker and Völker, 1990; Antelmann et al., 1996).

Regarding other groups of proteins over-synthesized in *S. oneidensis* at 30°C, we found GroEL (Spot 420), which increases the solubilization of aggregates (Niwa et al., 2012), the ClpB protein (Spot 152), and the DnaK (Spot 244), correlating the immunodetection results observed by WB. Protein GroEL belongs to the Hsp100/Clp AAA + ATPase family and cooperates with the chaperone system DnaK/DnaJ/GrpE in efficient solubilization and reactivation of aberrant protein aggregates formed as a consequence of cellular heat stress (Kedzierska et al., 2003; Mogk et al., 2003; Lee et al., 2004; Doyle et al., 2007). This chaperone restores the activity of other membrane proteins that also appeared over-synthesized in *S. oneidensis* at 30°C, the porin (spot 856), which forms hydrophilic channels in the membrane and is refolded by GroEL (Goulhen et al., 2004). Similar to *S. frigidimarina*, several other stress proteins over-accumulated could be indicative of a stress situation for *S. oneidensis* when grown at 30°C and/or due to be in the stationary phase. This is the case of universal stress protein (Usp) (Spot 1620). It is a small cytoplasmic protein that is greatly induced in many bacteria, such as *E. coli*, as a response to a wide range of environmental conditions and stressful situations like heat shock, starvation, or in the presence of stressors that prevent cell growth, and agents that damage DNA (Tkaczuk et al., 2013). This protein increases the survival rate of cells during prolonged exposure to these stressful conditions and is phosphorylated when cells enter the stationary growth phase (Sousa and McKay, 2001).

Again, the majority of proteins over-synthesized at 30°C in the case of the psychrophile *P. frigidicola* belong to the chaperone and stress protein groups. There were several of them involved in redox metabolism, resistance to oxidative stress or in heat stress, and the starvation response as it was observed for the other two bacteria studied. They were oxide and nitro-reductase enzymes that contribute to the defense against oxidative stress by reducing the redox cycle and the toxic nitro-aromatic compounds and quinones (Liochev et al., 1999), as well as degrading ROS and free radicals.

In this regard, two isoforms of the enzyme SOD (Spots 993 and 996), the protein thiorredoxin (Spot 1301) and DNA-Dps (Spot 1126), were induced in *P. frigidicola* at warm temperatures. The first protein plays a pivotal role in maintaining the redox state of the cell and protects it from oxidative stress (Wang et al., 2013; Lu and Holmgren, 2014). DNA-Dps, identified also in *S. frigidimarina*, is highly conserved and operates, as indicated before, against several kinds of stressors (Jeong et al., 2006; Karas et al., 2015) in *E. coli*, including oxidative damage to protect DNA. and it is also required in the normal starvation response and in long-term stationary viability (Almirón et al., 1992; Nair and Finkel, 2004).

Concerning the specific chaperones group, the main proteins found were involved in the heat stress response forming, as was the case for samples of *S. frigidimarina*, a cooperative role in the folding and maintenance of the proper conformation of other proteins to cope with environmental changes such as temperature.

### Comparative Molecular Machinery in Each Bacterium Against Temperature Conditions

Both temperature and oxidative stress are closely related, since temperature influences the solubility of oxygen and the formation of ROS (Piette et al., 2010; Chattopadhyay et al., 2011). Stress proteins were required at warm temperatures, whereas cold temperature seems to promote a similar membrane system in the two shewanellas studied.

The analysis of 2D-DIGE revealed common strategies in several groups of proteins between the three species, depending on growth temperature. The species *S. frigidimarina* shared over/under-synthesized proteins with *S. oneidensis* and *P. frigidicola*, while these two showed fewer proteins in common with one another. The strategy followed by *S. frigidimarina*, especially at warm temperatures, used shame chaperones and stress proteins with both *P. frigidicola* (GroES, TF, GrpE, ferritin, stationary phase protection protein) and *S. oneidensis* (ClpB, GroEL, and DnaK). On the other hand, the mesophile, *S. oneidensis*, and the psychrophile, *P. frigidicola*, employed different strategies at 30°C, except for the ClpB, which works together with DnaK to solubilize and reactivate aggregated proteins (Doyle et al., 2007; Doyle et al., 2015). This interaction is specie specific (Miot et al., 2011) and plays an essential role in cell survival under thermal stress conditions (Nagy et al., 2010; Krajewska et al., 2017).

Regarding oxidative stress, members of the superfamily of peroxiredoxins (peroxiredoxin C, thiorredoxin, and thiol peroxidase) were over-synthesized at 30°C in the two species of *Shewanella* and *Psychrobacter*. This kind of protein removes peroxides that are toxic to the cell (Storz and Imlay, 1999) and does also protects against osmotic stress and temperature changes in several bacteria (Leblanc et al., 2003). Even though oxidative stress is more typical at low temperatures (Chattopadhyay et al., 2011), it could also occur at 30°C as a response to the by-products of the increased metabolism of these bacteria at moderate to warm temperatures (Piette et al., 2010). Interestingly, the psychrotolerant *S. frigidimarina* may need to cope with oxidative stress at low temperatures through over-synthetization of other peroxiredoxin C isoforms. *S. oneidensis* showed a differential behavior in the synthesis of proteins related to the iron homeostasis. While it over-synthesized the non-heme ferritin at 4°C; the other two bacteria increased the levels of the Dps, which, as mentioned before, joins ferritin and protects against oxidative damage (Nair and Finkel, 2004; De Martino et al., 2016).

The metabolism of *S. oneidensis* and *S. frigidimarina* at low temperature, below their optimum, uses channels and siderophores that regulate the entrance of ions in the cell. These species at 4°C there induced synthesis of proteins of the outer membrane channels like TolPal System (TonB, TolC, and TolB) and the porin protein (that appears also in *P. frigidicola*), which interacts with this system for assembling to the membrane (Rigal et al., 1997). The system has an important role in the elimination of toxic compounds from oxidative damage through the membrane (Benz et al., 1993; Fralick, 1996) and its reparation (Zgurskaya et al., 2011) and binds to iron-chelating or siderophore complexes (Mirus et al., 2009). The porin protein, which was present in the three bacteria, could be acting differently in the psychrotolerant than in the other two species. It was over-synthesized at 4°C in *S. frigidimarina*, probably forming aqueous channels and interacting with the TolPal System (Cascales et al., 2002). However, it could be related to the basal apparatus of the flagellum (Chu et al., 2020; Liao et al., 2021) in *S. oneidensis*, in which it is over-synthesized at 30°C along with the flagellin protein.

Concerning replication and translation processes, there was an induction of different elongation factors like EF-G, EF-Tu, and EF-Ts, mainly at warm temperatures, except for factor EF-G in the mesophile and EF-Tu in the psychrophile. The EF-Ts is a guanine nucleotide exchanger for EF-Tu by GTPase activity for the separation of the EF-Tu-GDP complex from the ribosome (Palmer et al., 2013). It has a high similarity in different mesophiles such as *E. coli*, thermophiles such as *Thermus thermophilus*, and psychrophiles such as *Pseudoalteromonas haloplanktis*, but it was observed in higher concentration in the latter (Raimo et al., 2004). EF-G has different associations with ribosomes in response to stressful situations (deLivron et al., 2009). This factor is required for growth at low temperatures, as well as in acidic environments in *Sinorhizobium meliloti* (Kiss et al., 2004) and *E. coli* (Pfennig and Flower, 2001). It has also been described in other extremophiles such as *Psychrobacter cryohalolentis* and *Thiomicrospiracrunogena*, increasing the translation efficiency during the growth at low temperatures (Krishnan and Flower, 2008). Interestingly, EF-Tu that catalyzes the binding of aminoacyl-RNA to the ribosome can also act as a chaperone in the renaturation of other proteins (Masullo et al., 2000). This chaperone role has also been proposed for EF-Ts (Takeshita and Tomita, 2010) in the assembly and maintenance of the Qβ viral RNA polymerase in infected cells.

Regarding the groups of amino acids, nucleic acids, and proteins metabolism, there were no proteins identified that were shared by the three bacteria. Contrary to what has been indicated before for groups of stress and membrane proteins, no coincidences were found for these groups between *S. frigidimarina* and *P. frigidicola*, but both bacteria presented similarities with *S. oneidensis*. This could indicate that the main processes affected by stress and essential for cell survival and adaptation are those involving chaperone activity, detoxifying proteins, and transport through the membrane.

Several protein isoforms of different functional groups (peroxiredoxin C, DnaK, GroEL, EF-Tu, TonB-dependent receptor) present in *S. frigidimarina* were not observed in *S. oneidensis* or *P. frigidicola*. Other psychrotolerant species from Antarctica belonging to the genera *Sphingobacterium* and *Pseudomonas*, which can grow in the temperature range of 0–30°C, display differences in the phosphorylation-dephosphorylation state of membrane and cytosolic proteins in response to temperature changes, acting as cell heat sensors (Ray et al., 1994). The presence of isoforms in *S. frigidimarina* suggested that this psychrotolerant bacterium could have a similar mechanism to recognize environmental conditions and adapt to them, probably by different phosphorylation states or other post-translational modifications (dos Santos et al., 2010; García-Descalzo et al., 2014).

As a further step in understanding the molecular machinery that these three types of bacteria use to adapt to temperature changes, proteins that co-immunoprecipitate alongside the main chaperones DnaK and GroEL were investigated. Analysis of the results revealed protein interactions that could be happening *in vivo* in the cell as part of an adaptive response to different growth temperatures or as part of their normal metabolism at these temperatures (Figure 4).

Chaperones are essential components of the quality control machinery in cells. They optimize the physiological responses of bacteria by interacting directly with other proteins, taking part in cell signaling, and acting as stress response proteins (Hartl and Hayer-Hartl, 2002). Chaperones GroEL and DnaK are the main representatives of their families (Cooper and Hausman, 2000; Beckerman, 2005; Liberek et al., 2008), and their action has been described in a wide range of bacteria, frequently working together (Fourie and Wilson, 2020; Fatima et al., 2021; Mayer, 2021) and taking part in the regulation of transcription and translation (Parsot et al., 2003).

The molecular machinery involving these chaperones according to temperature was more complex in *S. oneidensis*, of intermediate complexity in *S. frigidimarina*, and notably simpler in *P. frigidicola*. While, in *S. oneidensis*, the proteins that co-immunoprecipitate with GroEL and DnaK tended to cluster at both warm and cold temperatures; those of *P. frigidicola* gathered mainly toward warmer temperatures, and *S. frigidimarina* showed a more equal distribution in the established and combined chaperone-temperature conditions. The groups of proteins that co-immunoprecipitated with the chaperones tested in *S. oneidensis* and *S. frigidimarina* shared more elements with each other than they do with proteins co-immunoprecipitated in *P. frigidicola*. These were proteins particularly involved in membrane transport, elongation processes, and oxidative stress. Characteristically, psychrotrophs are a more diverse group than psychrophiles, and their thermal properties overlap with those of some mesophiles (Russell, 1990). For *S. oneidensis*, a noticeably higher number of proteins seem to act together with GroEL at both temperatures and with DnaK at 30°C.

In general, the protein interaction network observed was mainly composed of chaperones and co-chaperones (GroES, Usp), membrane proteins involved in the transport of molecules and ions (porins and Ton-B-dependent receptors), and the formation of flagellar structures (flagellin, Omp family); but also proteins related with the defense against oxidative stress (peroxiredoxine, superoxide dismutase) and the elongation factors EF-Tu, EF-Ts, and EF-G. Co-immunoprecipitation revealed that the main processes to cope with temperature changes in these bacteria were those involved in the stress (temperature and oxidative) response. And that they need, especially *S. oneidensis*, the support of a membrane system that helps to eliminate toxic products resulting in these processes.

It is noteworthy that *P. frigidicola*, with a simpler proteome and a smaller number of proteins that co-immunoprecipitate with GroEL and Dnak than the two shewanellas, showed the co-appearance of the tellurium ion resistance (TerD) and SOD proteins with GroEL at both temperatures and with DnaK at 30°C for the former and DnaK at 4°C for the latter.

The TerD protein is part of a poorly characterized family involved especially in the response to oxidative stress in several organisms (Chasteen et al., 2009; Anantharaman et al., 2012; Daigle et al., 2015) and is also present in different pathogenic bacteria (Taylor, 1999; Turkovicova et al., 2016; Chen et al., 2019). The presence of this enzyme and SOD suggests that this psychrophilic bacterium is adapted to the frequent situation of oxidative stress at low temperatures due to the stability of ROS, and the metabolic processes that can lead to the generation of ROS at high temperatures (Piette et al., 2011).

## Conclusion

In the adaptation of microorganisms to changes in temperature, proteins are the molecular machine that regulates metabolism and cell functions. This study combines proteomic approaches with classical microbiology analysis, like growth dynamics.

We have observed a similar growing behavior between the three species against cold and warm temperatures, being able to achieve akin biomass under the temperatures tested, regardless of whether it is a stress situation or not. Some differences observed in the first stages of growth could give clues about whether the bacteria are in a normal or stressful situation, considering the duration of the lag phase. This observation had to be corroborated by a more detailed analysis, for which proteomic tools are essential.

The analysis of proteomic data showed that, in general, the two bacteria from the genus *Shewanella* displayed a more versatile adaptive response than the *Psychrobacter* species tested. These findings could suggest a tendency of an adaptation and evolution from mesophilic to psychrophilic microorganisms, maintaining a similar genome, but this would need to be supported with more genomic studies. Within the two *Shewanella* species studied, the psychrotolerant *S. frigidimarina* exhibits temperature-adaptative mechanisms in common with the other two species. This could be due to the genome (common with the mesophile) and the post-translational modifications, which give rise to isoforms (common with the psychrophile). As a result, it is better adapted to thermal variations in the temperature range 4–30°C.

In the molecular machinery to withstand temperature stressful situations, the role of chaperones is essential. In the three species studied, these proteins interact with other proteins forming complexes to provide diverse physiological responses of the cell to different growth temperatures. Equally important for adapting to temperature changes is the establishment of a cooperative system of chaperones with other proteins for the protection against oxidative damage, transmembrane transport, and elongation factors. They, together, are in charge of ensuring the viability and survival of bacteria.

This study is potentially related to pieces of climate change research since microorganisms and their adaptive strategies could be biosensors of the state of environments. An in-depth understanding of how they cope with changes in the temperature conditions could give us information about the molecules overproduced and provide a tool for evaluating the state of the environment. Additionally, it could also contribute to the Astrobiology field. The knowledge about the limits of life and how it adapts to environmental changes is crucial in the study of the habitability of environments with extreme conditions on Earth and in other planetary bodies like Mars or icy moons. The molecules employed to adapt to these extreme conditions could be considered biomarkers of life on Earth and beyond.

## Data Availability Statement

The mass spectrometry proteomics data have been deposited to the ProteomeXchange Consortium via the PRIDE repository with the dataset identifier PXD030438.

## Author Contributions

CC contributed to conceptualization, funding acquisition, editing, and manuscript review. LG-D contributed to experiments performance, analysis, and manuscript writing. EG-L contributed to experiments performance and data acquisition. All authors contributed to the article and approved the submitted version.

## Conflict of Interest

The authors declare that the research was conducted in the absence of any commercial or financial relationships that could be construed as a potential conflict of interest.

## Publisher’s Note

All claims expressed in this article are solely those of the authors and do not necessarily represent those of their affiliated organizations, or those of the publisher, the editors and the reviewers. Any product that may be evaluated in this article, or claim that may be made by its manufacturer, is not guaranteed or endorsed by the publisher.

## Footnotes

1
www.graphpad.com

## Abbreviations

SBd: substrate-binding domain-containing protein
TAXI-TRAP: TAXI family TRAP transporter solute-binding subunit
Omp: outer membrane porine
Phe-tRNA: phenylalanile-tRNA ligase subunit beta
RpiA: ribose-5-phosphate isomerase
cAMP-CRP: cAMP-activated global transcriptional regulator CRP protein
Flav-Sye4-NAD(P)H: flavin oxidoreductase Sye4
RpS1: ribosomal protein S1 30S
Pro-Iso: peptidyl-prolyl isomerase
TolC: outer membrane channel protein TolC
TonB: TonB-dependent siderophore receptor
GDHr: glutathione-disulfide reductase protein
PflB: formate C-acetyltransferase
PrpC: protein 2-methylcitrate synthase
Fur: ferric iron uptake transcriptional regulator
TolB: Tol-Pal system protein TolB
DH-E1: 2-oxoglutarate dehydrogenase E1 component
acyl-CoA DH: acyl-CoA dehydrogenase family protein
NpRibT: nicotinate phosphoribosyltransferase
CTP synthase: cytosine triphosphate synthase
PspA: phage shock protein A
ClpS: protease-dependent ATP adaptor protein Clp
Do-DegQ: periplasmic Do family serine endopeptidase DegQ
DNA-Dps: DNA starvation/stationary phase protection protein
Cyto-c-type: Chain A Tetraheme cytochrome c-type
Tpx-C: Thiol peroxidase protein subunit C
Q-nitroreductase -K: K subunit of the NADH quinone nitroreductase family protein.
