# Supplementary material for: Comparative Proteomic Analysis of Psychrophilic vs. Mesophilic Bacterial Species Reveals Different Strategies to Achieve Temperature Adaptation
Source: Front Microbiol. 2022 May 3;13:841359. doi: 10.3389/fmicb.2022.841359 (PMC9111180; doi:10.3389/fmicb.2022.841359)
Supplement: Supplementary file 1 [file Data_Sheet_1.docx]

Supplementary Material

# Supplementary Figures and Tables

**Supplementary Table 1 .** Identified proteins in *S. oneidendis*, *S. frigidimarina* and *P. frigidicola* that co-immunoprecipitate with GroEL at 4ºC.

| **Identified protein** | **Abbreviation** | **Specie** | **Score** | **Accession numb.** | **% SC** | **pI** | **M.M (Da)** | **Functional group** |
| --- | --- | --- | --- | --- | --- | --- | --- | --- |
| *S. oneidensis* | | | | | | | | |
| Peroxiredoxin C | PeroxC | *S. oneidensis* MR-1 | 132 | WP_011623136.1 | 75% | 5.01 | 22086 | Stress/chaperones |
| Superoxide dismutase [Fe] | SoD | *S. oneidensis* | 64 | WP_011072792.1 | 10% | 5.21 | 21539 | Stress/chaperones |
| GroEL | GroEL | *S. oneidensis* MR-1 | 231 | Q8CX48.1 | 73% | 4.84 | 57101 | Stress/chaperones |
| FKBP-type peptidyl-prolyl cis-trans isomerase | FKBP | *S. oneidensis* | 135 | WP_011071372.1 | 47% | 4.45 | 21855 | Stress/chaperones |
| TonB-dependent receptor | TonB | *S. oneidensis* | 170 | WP_011072403.1 | 93% | 4.38 | 92130 | Membrane/Transport |
| Porin | Por | *S. oneidensis* MR-1 | 248 | WP_011073642.1 | 72% | 4.7 | 39874 | Membrane/Transport |
| Outer membrane protein OmpK | OmpK | *S. oneidensis* MR-1 | 98 | WP_011071445 | 53% | 4.71 | 31430 | Membrane/Transport |
| Omp1/FadL family transporter | FadL | *S. oneidensis* MR-1 | 123 | WP_011072995.1 | 32% | 4.53 | 47034 | Membrane/Transport |
| Elongation factor G 2 | EF-G2 | *S. oneidensis* MR-1 | 207 | Q8EIJ7.1 | 70% | 4.91 | 77127 | Replic/Translat. |
| 30S ribosomal protein S1 | RpS1 | *S. oneidensis* | 155 | WP_011072381.1 | 57% | 4.91 | 61339 | Replic/Translat. |
| Elongation factor Tu | EF-Tu | *S. oneidensis* | 192 | WP_011070615.1 | 61% | 5.08 | 43542 | Replic/Translat. |
| Elongation factor Ts | EF-Ts | *S. oneidensis* | 80 | WP_011071785.1 | 43% | 5.31 | 30509 | Replic/Translat. |
| *S. frigidimarina* | | | | | | | | |
| Peroxiredoxin C | PeroxC | *Shewanella* | 132 | WP_011636928.1 | 21% | 5.22 | 22198 | Redox Homeost. |
| Porin | Por | *Shewanella* | 134 | WP_011635991.1 | 44% | 4.60 | 37708 | Membrane/Transport |
| TonB-dependent receptor | TonB | *Shewanella* | 87 | WP_011637870.1 | 22% | 4.19 | 90485 | Membrane/Transport |
| Elongation factor Tu | EF-Tu | *Shewanella* | 134 | WP_011635638.1 | 33% | 5.07 | 43547 | Replic/Translat. |
| dihydrolipoyl dehydrogenase | DHDL | *Shewanella* | 84 | WP_011639189.1 | 27% | 5.51 | 50860 | Aminoacid Metab. |
| *P, frigidicola* | | | | | | | | |
| Superoxido dismutase | SOD | *P. arcticus* | 140 | WP_011281195.1 | 34% | 5.03 | 23574 | Stress/chaperones |
| TerD family protein | TerD | *Psychrobacter* | 95 | WP_011280043.1 | 37% | 4.34 | 20425 | Stress/chaperones |
| GroEL | GroEL | **…** | **…** | **…** | **…** | **…** | **…** | Stress/chaperones |
| 50S ribosomal protein L6 | RpL6 | *P. arcticus* | 95 | WP_011279800.1 | 6% | 9.40 | 19510 | Replic/Translat. |

**Supplementary Table 2**. Identified proteins in *S. oneidendis*, *S. frigidimarina* and *P. frigidicola* that co-immunoprecipitate with GroEL at 30ºC.

| **GroEL 30°C** | | | | | | | | |
| --- | --- | --- | --- | --- | --- | --- | --- | --- |
| **Identified protein** | **Abbreviation** | **Specie** | **Score** | **Accession numb.** | **% SC** | **pI** | **M.M (Da)** | **Functional group** |
| *S. oneidensis* | | | | | | | | |
| Peroxiredoxin C | PeroxC | *Shewanella* | 253 | WP_011623136.1 | 34% | 5.01 | 22086 | Redox Homeost. |
| Universal stress protein | Usp | *S. oneidensis* | 136 | WP_011073476.1 | 79% | 5.26 | 15642 | Stress/chaperones |
| Chaperonin GroEL | GroEL | *S. oneidensis* | 273 | WP_011071022.1 | 56% | 4.84 | 57101 | Stress/chaperones |
| Two-component system response regulator ArcA | ArcA | *Shewanella* | 90 | WP_007644695.1 | 45% | 5.51 | 27260 | Stress/chaperones |
| TonB-dependent receptor | TonB | *S. oneidensis* | 215 | WP_011072815.1 | 35% | 4.54 | 95930 | Membrane/Transport |
| TonB-dependent receptor | TonB | *S. oneidensis* | 113 | WP_011072436.1 | 25% | 4.49 | 97928 | Membrane/Transport |
| Substrate-binding domain-containing protein | SBd | *Shewanella* | 188 | WP_011624595.1 | 66% | 6.47 | 29419 | Membrane/Transport |
| Flagellin | Fla | *S. oneidensis* | 130 | WP_011073121.1 | 49% | 7.90 | 28451 | Membrane/Transport |
| Substrate-binding domain-containing protein | SBd | *Shewanella* | 352 | WP_011074305.1 | 84% | 6.46 | 29348 | Membrane/Transport |
| TonB-dependent hemoglobin/transferrin/lactoferrin family receptor | TonBhtl | *S. oneidensis* | 112 | WP_011073464.1 | 38% | 4.80 | 76438 | Membrane/Transport |
| Ligand-gated channel protein | IrgA | *S. oneidensis* | 139 | WP_011074140.1 | 41% | 4.81 | 73259 | Membrane/Transport |
| TonB-dependent receptor | TonB | *S. oneidensi* | 197 | WP_011071669.1 | 42% | 4.81 | 90371 | Membrane/Transport |
| OmpP1/FadL family transporter | Omp1 | *S. oneidensis* | 171 | WP_011072995.1 | 59% | 4.53 | 47034 | Membrane/Transport |
| Porin | Por | *S. oneidensis* | 301 | WP_011073642.1 | 73% | 4.70 | 39874 | Membrane/Transport |
| Elongation factor Tu | EF-Tu | *S. oneidensis* | 313 | WP_011070604.1 | 76% | 5.13 | 43605 | Replic/Translat. |
| Elongation factor Ts | EF-Ts | *S. oneidensis* | 147 | WP_011071785.1 | 12% | 5.31 | 30509 | Replic/Translat. |
| L-threonine dehydrogenase | TDH | *S. oneidensis* | 150 | WP_011071675.1 | 50% | 5.68 | 40387 | Aminoacid Metab. |
| Glu/Leu/Phe/Val dehydrogenase | GLPVDH | *S. oneidensis* | 136 | WP_011072585.1 | 43% | 5.77 | 37343 | Aminoacid Metab. |
| Malate dehydrogenase | MDH | *S. oneidensis* | 137 | WP_011071073.1 | 47% | 5.37 | 32288 | Aminoacid Metab. |
| *S. frigidimarina* | | | | | | | | |
| Chaperonin GroEL | GroEL | *S. frigidimarina* | 211 | WP_011639075.1 | 53% | 4.83 | 57115 | Stress/chaperones |
| Superoxide dismutase [Fe] | SoD | *Shewanella* | 147 | WP_011637213.1 | 35% | 5.16 | 21621 | Stress/chaperones |
| TonB-dependent receptor | TonB | *S. frigidimarina* | 97 | WP_011637870.1 | 24% | 4.19 | 90485 | Membrane/Transport |
| Porin | Por | *S. frigidimarina* | 123 | WP_011635991.1 | 39% | 4.60 | 37708 | Membrane/Transport |
| Outer membrane protein OmpK | OmpK | *S. frigidimarina* | 134 | WP_011636475.1 | 15% | 4.62 | 31160 | Membrane/Transport |
| Elongation factor Tu | EF-Tu | *Shewanella* | 172 | WP_011635638.1 | 51% | 5.07 | 43547 | Replic/Translat. |
| *P, frigidicola* | | | | | | | | |
| TerD family protein | TerD | *Psychrobacter* | 85 | WP_011280043.1 | 39% | 4.36 | 20425 | Stress/chaperones |
| TerD family protein | TerD | *Psychrobacter* | 75 | WP_011959373.1 | 7% | 4.41 | 20381 | Stress/chaperones |
| Molecular chaperone DnaK | DnaK | *Psychrobacter* | 90 | WP_011961582.1 | 15% | 4.57 | 69094 | Stress/chaperones |
| GroEL | GroEL | *P. frigidicola* | 187 | B2ZRG7 | 28% | 4.75 | 57981 | Stress/chaperones |
| Superoxido dismutase | SoD | *P. arcticus* | 159 | WP_011281195.1 | 34% | 5.03 | 23574 | Stress/chaperones |
| Peptidylprolyl isomerase | Pro_Iso | *Psychrobacter* | 91 | WP_011279591.1 | 6% | 4.59 | 17415 | Replic/Translat. |

**Supplementary Table 3**. Identified proteins in *S. oneidendis*, *S. frigidimarina* and *P. frigidicola* that co-immunoprecipitate with DnaK at 4ºC.

| **DnaK 4°C** | | | | | | | | |
| --- | --- | --- | --- | --- | --- | --- | --- | --- |
| **Identified protein** | **Abbreviation** | **Specie** | **Score** | **Accession numb.** | **% SC** | **pI** | **M.M (Da)** | **Functional group** |
| *S. oneidensis* | | | | | | | | |
| Peroxiredoxin C | PeroxC | *Shewanella* | 140 | WP_011623136.1 | 18% | 5.01 | 22086 | Redox Homeost. |
| Universal stress protein | Usp | *S. oneidensis* | 166 | WP_011073476.1 | 32% | 5.26 | 15642 | Stress/chaperones |
| Co-chaperone GroES | GroES | *Shewanella* | 109 | WP_011071021.1 | 29% | 5.37 | 10207 | Stress/chaperones |
| PhageShockProteinA/IM30 family protein | PspA | *S. oneidensis* | 92 | WP_011073541.1 | 41% | 6.38 | 25374 | Stress/chaperones |
| Flagellin | Fla | *S. oneidensis* | 148 | WP_011073121.1 | 23% | 7.90 | 28451 | Membrane/Transport |
| OmpP1/FadL family transporter | Omp1 | *S. oneidensis* | 100 | WP_011072995.1 | 11% | 4.53 | 47034 | Membrane/Transport |
| Porin | Por | *S. oneidensis* | 239 | WP_011073642.1 | 17% | 4.70 | 39874 | Membrane/Transport |
| Substrate-binding domain-containing protein | SBd | *S. oneidensis* | 168 | WP_011074305.1 | 61% | 6.46 | 29348 | Membrane/Transport |
| Elongation factor Tu | EF-Tu | *S. oneidensis* | 212 | WP_011070604.1 | 31% | 5.13 | 43605 | Replic/Translat. |
| Glu/Leu/Phe/Val dehydrogenase | GLPVDH | *S. oneidensis* | 163 | WP_011072585.1 | 15% | 5.77 | 37343 | Aminoacid Metab. |
| Adenylate kinase | AdK | *S. oneidensis* | 111 | WP_011072102.1 | 25% | 5.57 | 23136 | Nucleotide Metab. |
| Nucleoside diphosphate kinase | NdK | *Shewanella* | 144 | WP_011072285.1 | 46% | 5.50 | 15530 | Nucleotide Metab. |
| L-threonine dehydrogenase | TDH | *S. oneidensis* | 120 | WP_011071675.1 | 17% | 5.68 | 40387 | Carbohydrate Metab. |
| CoA-acylating methylmalonate-semialdehyde dehydrogenase | MMSDH | *S. oneidensis* | 154 | WP_011071828.1 | 23% | 5.63 | 54157 | Carbohydrate Metab. |
| *S. frigidimarina* | | | | | | | | |
| TonB-dependent receptor | TonB | *S. frigidimarina* | 122 | WP_011637870.1 | 30% | 4.19 | 90485 | Membrane/Transport |
| TonB-dependent receptor | TonB | *S. frigidimarina* | 122 | WP_011636302.1 | 42% | 4.37 | 71830 | Membrane/Transport |
| Porin | Por | *Shewanella* | 168 | WP_011635991.1 | 43% | 4.60 | 37708 | Membrane/Transport |
| Elongation factor Tu | EF-Tu | *Shewanella* | 92 | WP_011635638.1 | 31% | 5.07 | 43547 | Replic/Translat. |
| Hypothetical protein | Hyp | *S. frigidimarina* | 310 | WP_011636938.1 | 34% | 5.13 | 41434 | Hypothetical |
| *P, frigidicola* | | | | | | | | |
| Superoxide dismutase | SoD | *Psychrobacter* | 73 | WP_011959598.1 | 33% | 4.97 | 23481 | Stress/chaperones |
| DnaK |  | … | … | … | … | … | … | Stress/chaperones |

**Supplementary Table 4**. Identified proteins in *S. oneidendis*, *S. frigidimarina* and *P. frigidicola* that co-immunoprecipitate with DnaK at 30ºC.

| **DnaK 30°C** | | | | | | | | |
| --- | --- | --- | --- | --- | --- | --- | --- | --- |
| **Identified protein** | **Abbreviation** | **Specie** | **Score** | **Accession numb.** | **% SC** | **pI** | **M.M (Da)** | **Functional group** |
| *S. oneidensis* | | | | | | | | |
| DnaK |  | *…* | *…* | *…* | *…* | *…* | *…* | Stress/chaperones |
| Substrate-binding domain-containing protein | SBd | *S. putrefaciens* CN-32 | 212 | WP_011920275.1 | 31% | 6.15 | 29511 | Membrane/Transport |
| Substrate-binding domain-containing protein | SBd | *S. oneidensis* | 339 | WP_011074305.1 | 61% | 6.46 | 29348 | Membrane/Transport |
| Porin | Por | *S. oneidensis* | 227 | WP_011073642.1 | 72% | 4.70 | 39874 | Membrane/Transport |
| Flagellin | Fla | *S. oneidensis* | 103 | WP_011073121.1 | 35% | 7.90 | 28451 | Membrane/Transport |
| Elongation factor Tu | EF-Tu | *S. oneidensis* | 128 | WP_011070604.1 | 33% | 5.13 | 43605 | Replic/Translat. |
| *S. frigidimarina* | | | | | | | | |
| Peroxiredoxin C | PeroxC | *Shewanella* | 105 | WP_011636928.1 | 28% | 5.22 | 22198 | Redox Homeost. |
| Superoxide dismutase [Fe] | SoD | *Shewanella* | 124 | WP_011637213.1 | 41% | 5.16 | 21621 | Stress/chaperones |
| Porin | Por | *Shewanella* | 92 | WP_011635991.1 | 45% | 4.60 | 37708 | Membrane/Transport |
| Elongation factor Tu | EF-Tu | *Shewanella* | 147 | WP_011635638.1 | 52% | 5.07 | 43547 | Replic/Translat. |
| Ribosome recycling factor | Rrc | *Shewanella* | 95 | WP_011636747.1 | 47% | 5.27 | 20646 | Replic/Translat. |
| Hypothetical protein | Hyp | *Psychrobacter* | 89 | WP_011513379.1 |  |  | 24558 | Hypothetical |
| *P, frigidicola* | | | | | | | | |
| TerD family protein | TerD | *Psychrobacter* | 71 | WP_011959373.1 | 7% | 4.41 | 20381 | Stress/chaperones |
| TerD family protein | TerD | *Psychrobacter* | 103 | WP_011280043.1 | 6% | 4.36 | 20425 | Stress/chaperones |
| ATP-dependent chaperone ClpB | ClpB | *P. sanguinis* | 90 | WP_007393820.1 | 13% | 5.06 | 96057 | Stress/chaperones |
| DnaK | DnaK | Psychrobacter sp. PAMC 21119 | 70 | ZP_10789086 | 10% | 4.50 | 69456 | Stress/chaperones |
| GroEL | GroEL | *P. frigidicola* | 82 | ACD68572 | 11% | 4.75 | 57981 | Stress/chaperones |
| TonB-dependent receptor | TonB | *Psychrobacter sp.* PRwf-1 | 84 | WP_011960727.1 | 2% | 4.94 | 81639 | Membrane/Transport |

## Supplementary Figures


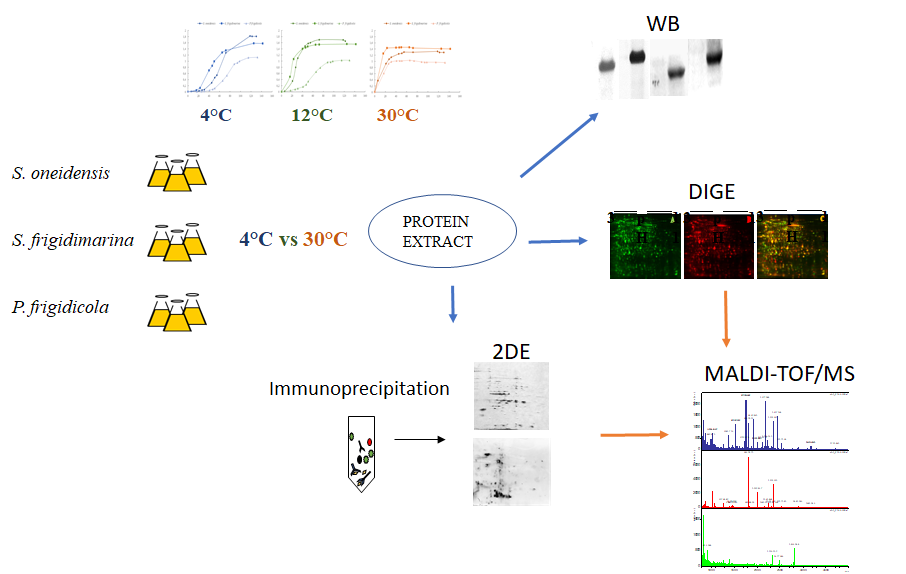


**Supplementary Figure 1.** Summary of the overall experimental strategy. Triplicates of cultures of the three bacteria at different temperatures: Growth rate of samples at 4ºC, 12ºC and 30ºCwere monitored by optical density at 600nm. Proteins were extracted from cultures at 4ºC and 30ºC to be compared in: (i) western-blot with anti-DnaK, anti-DnaJ, anti-GroEL and anti-GroES antibodies; (ii) 2D-DIGE experiments and (iii) 2DE from extracts immunoprecipitated with anti-DnaK and anti-GorEL antibodies. Proteins from DIGE and 2DE experiments were identified by MALDI-TOF/MS.


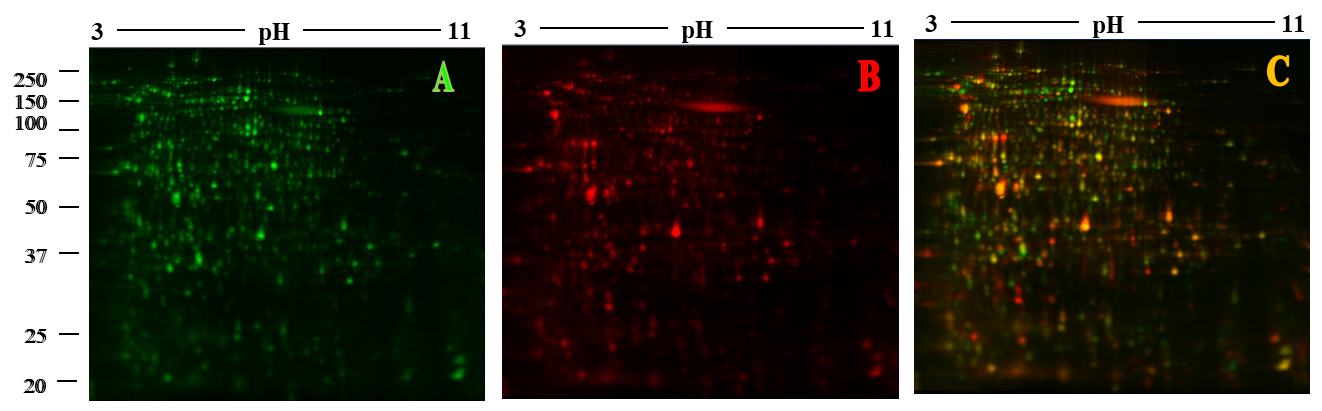


**Supplementary Figure 2.** Images of two-dimensional fluorescent gels for 2D-DIGE comparison of *Shewanella oneidensis* total protein extracts from cultures at 4ºC and 30ºC. 24 cm non-linear pH 3-11 IPG strips and a molecular weight marker shown on the left were used. (A) Gel with samples from 4°C condition labelled with Cy3 fluorochrome and excited at the corresponding wavelength; (B) samples from 30°C condition labelled with Cy5 fluorochrome, excited at the corresponding wavelength; (C) The overlay of the two previous images, noting the differences in the synthesis levels of the protein spots that appear in this image as a result of exciting both fluorochromes at the same time.


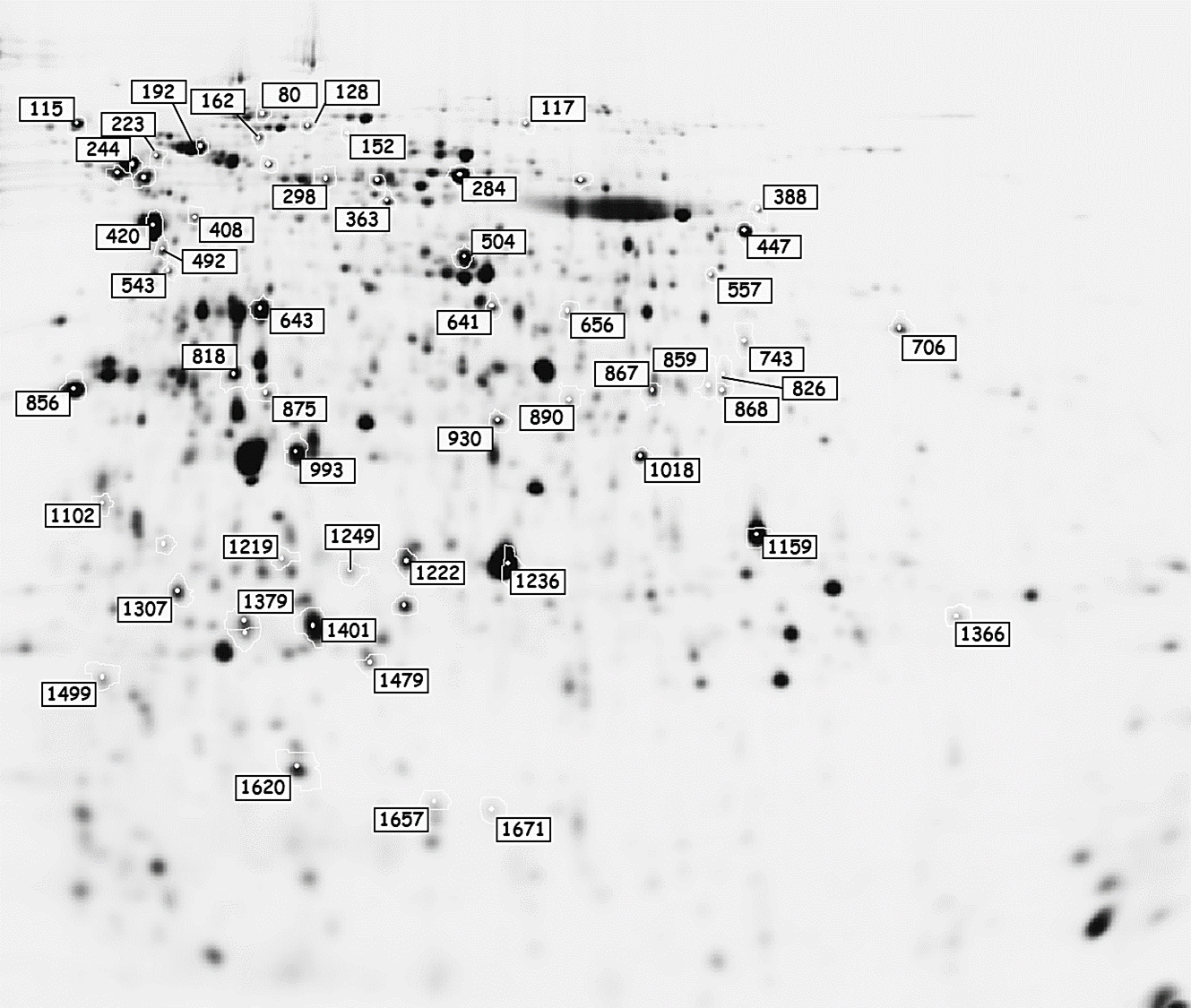


**Supplementary Figure 3.** Representative image of a 2D gel of the total protein extract of *Shewanella oneidensis* showing protein spots with statistically significant differences in the comparative analysis of extracts at 4°C and 30°C, which were cut out of the gel, trypsinised and identified by mass spectrometry**.** Spots are numbered according to the corresponding table 5.


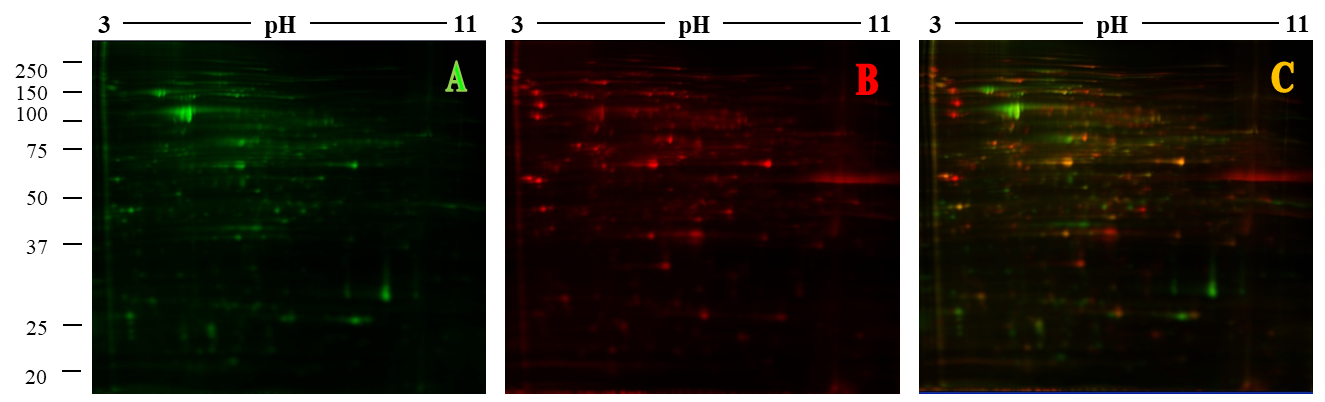


**Supplementary Figure 4.** Images of two-dimensional fluorescent gels for 2D-DIGE comparison of *Shewanella frigidimarina* total protein extracts from cultures at 4ºC and 30ºC. 24 cm non-linear pH 3-11 IPG strips and a molecular weight marker shown on the left were used. (A) Gel with samples from 4°C condition labelled with Cy3 fluorochrome and excited at the corresponding wavelength; (B) samples from 30°C condition labelled with Cy5 fluorochrome, excited at the corresponding wavelength; (C) The overlay of the two previous images, noting the differences in the synthesis levels of the protein spots that appear in this image as a result of exciting both fluorochromes at the same time.


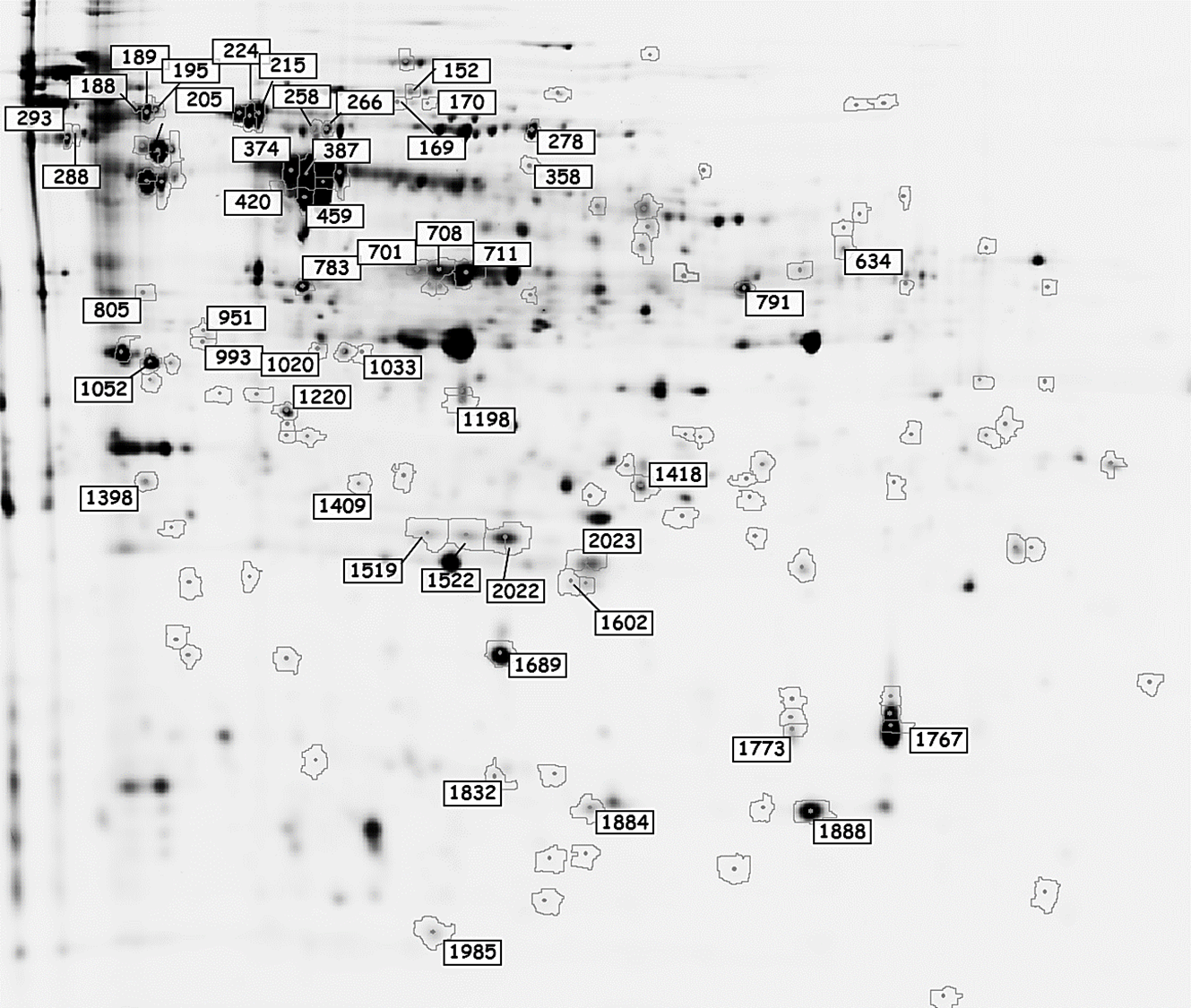


**Supplementary Figure 5.** Representative image of a 2D gel of the total protein extract of *Shewanella frigidimarina* showing protein spots with statistically significant differences in the comparative analysis of extracts at 4°C and 30°C, which were cut out of the gel, trypsinised and identified by mass spectrometry**.** Spots are numbered according to the corresponding table 6.


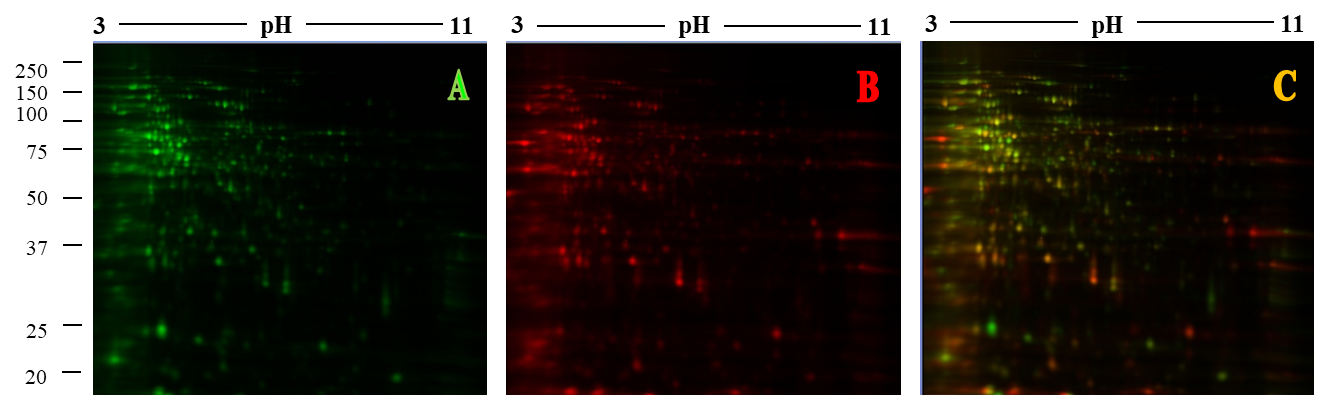


**Supplementary Figure 6.** Images of two-dimensional fluorescent gels for 2D-DIGE comparison of *Psychrobacter frigidicola* total protein extracts from cultures at 4ºC and 30ºC. 24 cm non-linear pH 3-11 IPG strips and a molecular weight marker shown on the left were used. (A) Gel with samples from 4°C condition labelled with Cy3 fluorochrome and excited at the corresponding wavelength; (B) samples from 30°C condition labelled with Cy5 fluorochrome, excited at the corresponding wavelength; (C) The overlay of the two previous images, noting the differences in the synthesis levels of the protein spots that appear in this image as a result of exciting both fluorochromes at the same time.


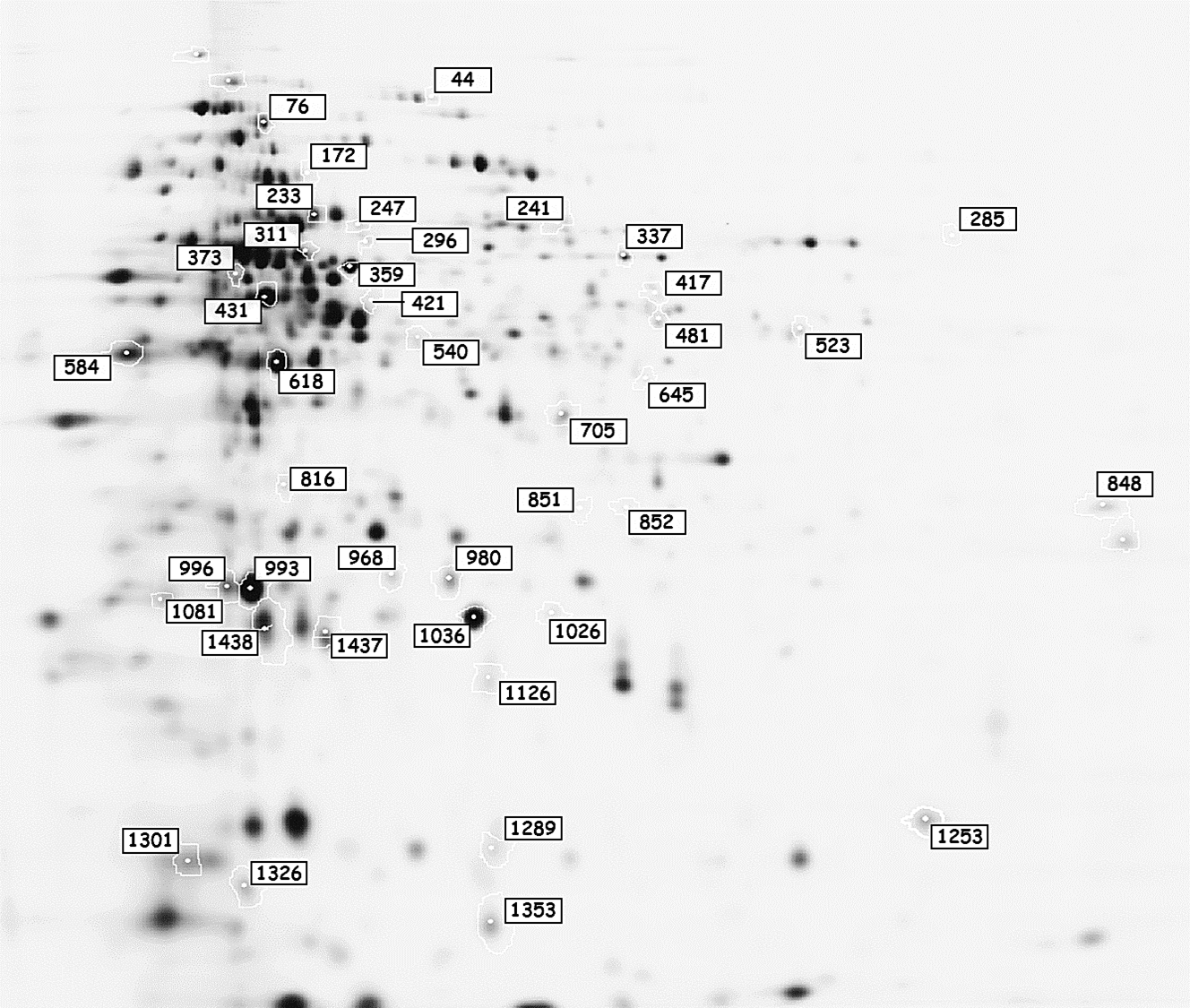


**Supplementary Figure 7.** Representative image of a 2D gel of the total protein extract of *Psychrobacter frigidicola* showing protein spots with statistically significant differences in the comparative analysis of extracts at 4°C and 30°C, which were cut out of the gel, trypsinised and identified by mass spectrometry**.** Spots are numbered according to the corresponding table 7.

# Supplementary Discussion

3.1. Over-synthesised proteins at low temperature

In S*. frigidimarina* growing at 4°C there was a high accumulation of membrane transport proteins and proteins related to iron metabolism and oxidative stress but another pathway that appeared to be affected by temperature in this specie, entails the agmatine deiminase system (spot 951) that participates in stress tolerance. It has been reported that this system plays an important role in tolerance to the acidic environment in *Listeria monocytogenes* (Chen et al., 2011).

We found in cultures of *S. oneidensis* at 4°C an increase in redox enzymes like Flav-Sye4 (spot 875). Induction studies of this family of enzymes in *S. oneidensis* reveal their involvement in the protection of cell components against oxidative stress (Brigé et al., 2006). Furthermore, the metabolism of iron is well represented in these samples and showed an over- synthesis of (i) the ferric iron uptake transcriptional regulator (Fur) (spot 1671), involved in iron acquisition, acidity tolerance, response to oxidative stress, and flagella chemotaxis (Yang et al., 2008); as a global regulator of iron homeostasis it controls the absorption of iron under limiting conditions, and the synthesis of iron-storing proteins and iron-using enzymes, when iron levels are high (Lee and Helmann, 2007); (ii) non-heme ferritin (spot 1499), from the Dps family, that protects bacteria from many stresful situations like starvation, heat shock, overexposure to iron (Nair and Finkel, 2004; Karas et al., 2015) and oxidative stress (Smith, 2004) by drawing iron to avoid the production of toxic free radicals, especially during the stationary growth phase (Calhoun and Kwon, 2011); (iii) the SidA/IucD/PvdA family monooxygenase (spot 656) which is an iron chelant or siderophore (Olucha and Lamb, 2011); (iv). Among them, the Ton-B, a member of the porine superfamily, achieved the highest ratio (12.7). It is an external membrane receptor protein that binds to iron-chelating or siderophore complexes (Mirus et al., 2009). Ton-B provides the necessary energy for the translocation of the siderophore complexes through the membrane (Andrews et al., 2003) which are secreted under iron-limiting conditions (Ferreira and Straus, 1994). The TolB complex interacts with the porins for their assembly to the cell membrane (Rigal et al., 1997). TolC enables the transport of various toxic molecules, potentially generated as a consequence of the oxidative stress, across the outer membrane (Benz et al., 1993; Fralick, 1996), contributing to detoxification and repair after oxidative stress (Zgurskaya et al., 2011).

Specifically and in relation to the metabolic path that appeared to be triggered in at 4°C in *S. oneidensis* related with intracellular levels of acetyl-CoA, we found an increase in the synthesis of the (i) PflB (spot 298) that enhance levels of acetyl-CoA in the cell (Wagner et al., 1992) and plays a central role in the anaerobic metabolism of *E. coli* producing formate and acetyl-CoA by the non-oxidative route (Nnyepi et al., 2007); (ii) PrpC (spot 867) which turns the propionate to pyruvate in the oxidation cycle (Claes et al., 2002) and has both acetyl-CoA and propionyl-CoA as substrates (Gerike et al., 1998); and (iii) the acetate kinase (spot 743), a protein that controls the cell concentration of acetyl-CoA (Ingram-Smith et al., 2006) and acetyl-phosphate, which are both essential secondary metabolites (Chittori et al., 2012).

The active metabolism detected at 4ºC in the case of *P. frigidicola* was represented by the increase of proteins involved in different metabolic paths of biosybthesis of aminoacids, nucleotides and carbohydrates, like the 2-oxoglutarate dehydrogenase E1 component (spot 44), arginosuccinate synthase (spot 373), glutamate 5-kinase (spot 421), CTP synthase (spot 247), and acyl-CoA dehydrogenase family protein (spot 481). In addition, several factors and proteins involved in the synthesis and translation of other proteins were also induced in this bacterium at 4ºC: NpRibT (spot 417), the alpha-subunit of Phe-RS (spot 540), the EF-Tu (spot 431) and the translational GTPase TypA TypA (also called BipA) (spot 172), that forms the machinery necessary for protein translation, and is also a cold shock–inducible GTPase (Choi and Hwang, 2018).

3.2. Over-synthesized proteins at high temperature

An important group of proteins extensively represented in *S. frigidimarina* at 30 ºC was related with synthesis, including several GTPases, elongation factors, and ribosomal proteins. The high production of these proteins in *S. frigidimarina* could represent an adaptation tool to ensure the efficiency of nucleotide exchange even in stressful situations (Sprinzl, 2000). In our experiments we found two isoforms of the ribosomal protein S1 30S (spots 266 and 258), three of EF-Tu (spots 701, 708 and 711), EF-G (spot 152), EF-Ts (spot 1198), and tRNA uridine-5-carboxymethylaminomethyl (34) synthesis GTPase MnmE (spot 459).

Besides different chaperones, other proteins facing stress situations were also increased in *S. frigidimarina* at 30°C: (i) PspA (spot 1418) that participates in maintaining the integrity of the cytoplasmic membrane under different stressful conditions including thermal stress (Darwin, 2005); (ii) ClpS (spot 1884) that is involved in protein degradation by providing substrates to the chaperone proteinase pair system ClpAP (Román-Hernández et al., 2011), which degrades aberrant protein aggregates formed as a result of increased temperature (Dougan et al., 2002); and (iii) Do-DegQ (spot 791), a serin-protease belonging to the highly conserved family HtrA, that regulates proteolysis by ATP-independent quality control and can act as a chaperone too (Clausen et al., 2011).

In relation with oxidative stress, in addition to proteins from Dps and peroxiredoxine families, the Cyto-c-type (spot 1985) was also identified. It is a periplasmic tetraheme protein with catalytic fumarate reductase activity widely studied in *S. frigidimarina* (Turner et al., 1999).

In the case of S. oneidensis at 30ºC and among over accumulated proteins related to membrane transport, we found flagellin (spot 993), iron (III) ABC transporter (spot 930), substrate-binding domain-containing protein (spot 1236) and TAXI-TRAP (spot 1018), the Omp (spot 856). Similar to *S. firigidimarina* the Do-DegQ protein (spot 641), was also induced in these samples. This protein has serine protease and chaperone activities. It is involved in the refolding and degradation of damaged proteins, and is considered as an ATP-independent chaperone-protease (Kim and Kim, 2005). This proteolytic function is predominant at high temperatures (HtrA – required at high temperature) while at low temperatures it mainly acts as a chaperone, whose activity resides in the PDZ domains (Krojer et al., 2002; Kim et al., 2003; Kim and Kim, 2005; Clausen et al., 2011).

In relation with redox metabolisms there were also an increase in the synthesis of the Q-nitroreductase-K (spot 1289), the uroporphyrinogen decarboxylase (spot 645), the nitroreductase family protein (spot 1036), and the vicinal oxygen chelate (VOC) family (spot 980). Studies carried out on *E. coli* indicate that the glyoxalase (VOC family protein), with a dioxygenase domain and an active metal-binding center, plays an important role in the detoxification pathway of eukaryotes, fungi and bacteria (Sukdeo et al., 2004). In some prokaryotes this enzyme protects against the cell damage caused on proteins and nucleic acids by methylglyoxylate that is generated when there is a shortage of phosphorus (Vickers et al., 2004).

An extense list of proteins against stress were identified with synthesis increased in cultures of *P. frigidimarina* at 30°C like the trigger factor (TF) (spot 359), GrpE protein (spot 1081), GroES chaperonin (spot 1326), a Pro-Iso (spot 1438) and the ClpB (spot 76). These proteins are closely related to each other to perform a cellular response against aberrant aggregates of proteins formed by a stress condition. The peptidyl-prolyl isomerase is a cilophylline that accelerates protein folding by catalyzing proline isomerization in peptide bonds (Stamnes et al., 1992) and it can also act as a chaperone (Wang and Heitman, 2005). The ATP-dependent chaperone ClpB belongs to the family of ATPases associated with diverse cell activities (AAA^+^ ATPases). This protein can function as a chaperone in many organisms (Ogura and Wilkinson, 2001; Iyer et al., 2004; Snider et al., 2008; Biter et al., 2012) by degrading aggregates and stabilization protein folding, and frequently cooperates with DnaK chaperone (Mogk et al., 1999; Zolkiewski, 1999; Lee et al., 2003; Krajewska et al., 2017). Other heat shock proteins and proteases belonging to this AAA^+^ ATPases family, including the subfamily Clp/Hsp100 (Lee et al., 2004) were identified. They, like ClpB, often interact with other chaperones of the Hsp70/Hsp40 family (Sielaff and Tsai, 2010) to which the GrpE is cofactor (Langer et al., 1992) and responsible for the exchange of ADP by ATP (Bonomo et al., 2010). In turn, it cooperates with the system formed by GroEL/GroES and TF, widely described in *P. frigidicola*, where it also exhibits a peptidyl-prolyl isomerase activity (Robin et al., 2009), and in *P. haloplanktis* TAC125, where it shows a strong dependence on the temperature being induced at moderately warm temperatures (18ºC-33ºC) (Piette et al., 2010). Nevertheless, in *P. frigidicola* TF, unlike in other bacteria, is a monomeric chaperone that could act independently from other chaperones (Robin et al., 2009).

In addition to stress proteins, there was a substantial group of proteins related to aminoacid metabolism, especially peptidases and aminotransferases that regulate the cell exchange of proteins, and whose synthesis is increased at 30ºC in *P. frigidicola.*

**Supplementary References**

Andrews, S.C., Robinson, A.K., and Rodríguez-Quiñones, F. (2003). Bacterial iron homeostasis. *FEMS Microbiol Rev* 27(2-3)**,** 215-237. doi: 10.1016/s0168-6445(03)00055-x.

Benz, R., Maier, E., and Gentschev, I. (1993). TolC of Escherichia coli functions as an outer membrane channel. *Zentralbl Bakteriol* 278(2-3)**,** 187-196. doi: 10.1016/s0934-8840(11)80836-4.

Biter, A.B., Lee, J., Sung, N., Tsai, F.T., and Lee, S. (2012). Functional analysis of conserved cis- and trans-elements in the Hsp104 protein disaggregating machine. *J Struct Biol* 179(2)**,** 172-180. doi: 10.1016/j.jsb.2012.05.007.

Bonomo, J., Welsh, J.P., Manthiram, K., and Swartz, J.R. (2010). Comparing the functional properties of the Hsp70 chaperones, DnaK and BiP. *Biophys Chem* 149(1-2)**,** 58-66. doi: 10.1016/j.bpc.2010.04.001.

Brigé, A., Van den Hemel, D., Carpentier, W., De Smet, L., and Van Beeumen, J.J. (2006). Comparative characterization and expression analysis of the four Old Yellow Enzyme homologues from Shewanella oneidensis indicate differences in physiological function. *Biochem J* 394(Pt 1)**,** 335-344. doi: 10.1042/bj20050979.

Calhoun, L.N., and Kwon, Y.M. (2011). Structure, function and regulation of the DNA-binding protein Dps and its role in acid and oxidative stress resistance in Escherichia coli: a review. *J Appl Microbiol* 110(2)**,** 375-386. doi: 10.1111/j.1365-2672.2010.04890.x.

Chen, J., Cheng, C., Xia, Y., Zhao, H., Fang, C., Shan, Y., et al. (2011). Lmo0036, an ornithine and putrescine carbamoyltransferase in Listeria monocytogenes, participates in arginine deiminase and agmatine deiminase pathways and mediates acid tolerance. *Microbiology (Reading)* 157(Pt 11)**,** 3150-3161. doi: 10.1099/mic.0.049619-0.

Chittori, S., Savithri, H.S., and Murthy, M.R. (2012). Structural and mechanistic investigations on Salmonella typhimurium acetate kinase (AckA): identification of a putative ligand binding pocket at the dimeric interface. *BMC Struct Biol* 12**,** 24. doi: 10.1186/1472-6807-12-24.

Choi, E., and Hwang, J. (2018). The GTPase BipA expressed at low temperature in Escherichia coli assists ribosome assembly and has chaperone-like activity. *J Biol Chem* 293(47)**,** 18404-18419. doi: 10.1074/jbc.RA118.002295.

Claes, W.A., Pühler, A., and Kalinowski, J. (2002). Identification of two prpDBC gene clusters in Corynebacterium glutamicum and their involvement in propionate degradation via the 2-methylcitrate cycle. *J Bacteriol* 184(10)**,** 2728-2739. doi: 10.1128/jb.184.10.2728-2739.2002.

Clausen, T., Kaiser, M., Huber, R., and Ehrmann, M. (2011). HTRA proteases: regulated proteolysis in protein quality control. *Nat Rev Mol Cell Biol* 12(3)**,** 152-162. doi: 10.1038/nrm3065.

Darwin, A.J. (2005). The phage-shock-protein response. *Mol Microbiol* 57(3)**,** 621-628. doi: 10.1111/j.1365-2958.2005.04694.x.

Dougan, D.A., Reid, B.G., Horwich, A.L., and Bukau, B. (2002). ClpS, a substrate modulator of the ClpAP machine. *Mol Cell* 9(3)**,** 673-683. doi: 10.1016/s1097-2765(02)00485-9.

Ferreira, F., and Straus, N.A. (1994). Iron deprivation in cyanobacteria. *Journal of Applied Phycology* 6(2)**,** 199-210. doi: 10.1007/BF02186073.

Fralick, J.A. (1996). Evidence that TolC is required for functioning of the Mar/AcrAB efflux pump of Escherichia coli. *J Bacteriol* 178(19)**,** 5803-5805. doi: 10.1128/jb.178.19.5803-5805.1996.

Gerike, U., Hough, D.W., Russell, N.J., Dyall-Smith, M.L., and Danson, M.J. (1998). Citrate synthase and 2-methylcitrate synthase: structural, functional and evolutionary relationships. *Microbiology (Reading)* 144 ( Pt 4)**,** 929-935. doi: 10.1099/00221287-144-4-929.

Ingram-Smith, C., Martin, S.R., and Smith, K.S. (2006). Acetate kinase: not just a bacterial enzyme. *Trends Microbiol* 14(6)**,** 249-253. doi: 10.1016/j.tim.2006.04.001.

Iyer, L.M., Leipe, D.D., Koonin, E.V., and Aravind, L. (2004). Evolutionary history and higher order classification of AAA+ ATPases. *J Struct Biol* 146(1-2)**,** 11-31. doi: 10.1016/j.jsb.2003.10.010.

Karas, V.O., Westerlaken, I., and Meyer, A.S. (2015). The DNA-Binding Protein from Starved Cells (Dps) Utilizes Dual Functions To Defend Cells against Multiple Stresses. *J Bacteriol* 197(19)**,** 3206-3215. doi: 10.1128/jb.00475-15.

Kim, D.Y., Kim, D.R., Ha, S.C., Lokanath, N.K., Lee, C.J., Hwang, H.Y., et al. (2003). Crystal structure of the protease domain of a heat-shock protein HtrA from Thermotoga maritima. *J Biol Chem* 278(8)**,** 6543-6551. doi: 10.1074/jbc.M208148200.

Kim, D.Y., and Kim, K.K. (2005). Structure and function of HtrA family proteins, the key players in protein quality control. *J Biochem Mol Biol* 38(3)**,** 266-274. doi: 10.5483/bmbrep.2005.38.3.266.

Krajewska, J., Modrak-Wójcik, A., Arent, Z.J., Więckowski, D., Zolkiewski, M., Bzowska, A., et al. (2017). Characterization of the molecular chaperone ClpB from the pathogenic spirochaete Leptospira interrogans. *PLoS One* 12(7)**,** e0181118. doi: 10.1371/journal.pone.0181118.

Krojer, T., Garrido-Franco, M., Huber, R., Ehrmann, M., and Clausen, T. (2002). Crystal structure of DegP (HtrA) reveals a new protease-chaperone machine. *Nature* 416(6879)**,** 455-459. doi: 10.1038/416455a.

Langer, T., Lu, C., Echols, H., Flanagan, J., Hayer, M.K., and Hartl, F.U. (1992). Successive action of DnaK, DnaJ and GroEL along the pathway of chaperone-mediated protein folding. *Nature* 356(6371)**,** 683-689. doi: 10.1038/356683a0.

Lee, J.W., and Helmann, J.D. (2007). Functional specialization within the Fur family of metalloregulators. *Biometals* 20(3-4)**,** 485-499. doi: 10.1007/s10534-006-9070-7.

Lee, S., Sowa, M.E., Choi, J.M., and Tsai, F.T. (2004). The ClpB/Hsp104 molecular chaperone-a protein disaggregating machine. *J Struct Biol* 146(1-2)**,** 99-105. doi: 10.1016/j.jsb.2003.11.016.

Lee, S., Sowa, M.E., Watanabe, Y.H., Sigler, P.B., Chiu, W., Yoshida, M., et al. (2003). The structure of ClpB: a molecular chaperone that rescues proteins from an aggregated state. *Cell* 115(2)**,** 229-240. doi: 10.1016/s0092-8674(03)00807-9.

Mirus, O., Strauss, S., Nicolaisen, K., von Haeseler, A., and Schleiff, E. (2009). TonB-dependent transporters and their occurrence in cyanobacteria. *BMC Biol* 7**,** 68. doi: 10.1186/1741-7007-7-68.

Mogk, A., Tomoyasu, T., Goloubinoff, P., Rüdiger, S., Röder, D., Langen, H., et al. (1999). Identification of thermolabile Escherichia coli proteins: prevention and reversion of aggregation by DnaK and ClpB. *Embo j* 18(24)**,** 6934-6949. doi: 10.1093/emboj/18.24.6934.

Nair, S., and Finkel, S.E. (2004). Dps protects cells against multiple stresses during stationary phase. *Journal of bacteriology* 186(13)**,** 4192-4198. doi: 10.1128/JB.186.13.4192-4198.2004.

Nnyepi, M.R., Peng, Y., and Broderick, J.B. (2007). Inactivation of E. coli pyruvate formate-lyase: role of AdhE and small molecules. *Arch Biochem Biophys* 459(1)**,** 1-9. doi: 10.1016/j.abb.2006.12.024.

Ogura, T., and Wilkinson, A.J. (2001). AAA+ superfamily ATPases: common structure--diverse function. *Genes Cells* 6(7)**,** 575-597. doi: 10.1046/j.1365-2443.2001.00447.x.

Olucha, J., and Lamb, A.L. (2011). Mechanistic and structural studies of the N-hydroxylating flavoprotein monooxygenases. *Bioorganic chemistry* 39(5-6)**,** 171-177. doi: 10.1016/j.bioorg.2011.07.006.

Piette, F., D'Amico, S., Struvay, C., Mazzucchelli, G., Renaut, J., Tutino, M.L., et al. (2010). Proteomics of life at low temperatures: trigger factor is the primary chaperone in the Antarctic bacterium Pseudoalteromonas haloplanktis TAC125. *Mol Microbiol* 76(1)**,** 120-132. doi: 10.1111/j.1365-2958.2010.07084.x.

Rigal, A., Bouveret, E., Lloubes, R., Lazdunski, C., and Benedetti, H. (1997). The TolB protein interacts with the porins of Escherichia coli. *J Bacteriol* 179(23)**,** 7274-7279. doi: 10.1128/jb.179.23.7274-7279.1997.

Robin, S., Togashi, D.M., Ryder, A.G., and Wall, J.G. (2009). Trigger factor from the psychrophilic bacterium Psychrobacter frigidicola is a monomeric chaperone. *J Bacteriol* 191(4)**,** 1162-1168. doi: 10.1128/jb.01137-08.

Román-Hernández, G., Hou, J.Y., Grant, R.A., Sauer, R.T., and Baker, T.A. (2011). The ClpS adaptor mediates staged delivery of N-end rule substrates to the AAA+ ClpAP protease. *Mol Cell* 43(2)**,** 217-228. doi: 10.1016/j.molcel.2011.06.009.

Sielaff, B., and Tsai, F.T. (2010). The M-domain controls Hsp104 protein remodeling activity in an Hsp70/Hsp40-dependent manner. *J Mol Biol* 402(1)**,** 30-37. doi: 10.1016/j.jmb.2010.07.030.

Smith, J.L. (2004). The physiological role of ferritin-like compounds in bacteria. *Crit Rev Microbiol* 30(3)**,** 173-185. doi: 10.1080/10408410490435151.

Snider, J., Thibault, G., and Houry, W.A. (2008). The AAA+ superfamily of functionally diverse proteins. *Genome Biol* 9(4)**,** 216. doi: 10.1186/gb-2008-9-4-216.

Stamnes, M.A., Rutherford, S.L., and Zuker, C.S. (1992). Cyclophilins: a new family of proteins involved in intracellular folding. *Trends Cell Biol* 2(9)**,** 272-276. doi: 10.1016/0962-8924(92)90200-7.

Sukdeo, N., Clugston, S.L., Daub, E., and Honek, J.F. (2004). Distinct classes of glyoxalase I: metal specificity of the Yersinia pestis, Pseudomonas aeruginosa and Neisseria meningitidis enzymes. *Biochem J* 384(Pt 1)**,** 111-117. doi: 10.1042/bj20041006.

Turner, K.L., Doherty, M.K., Heering, H.A., Armstrong, F.A., Reid, G.A., and Chapman, S.K. (1999). Redox properties of flavocytochrome c3 from Shewanella frigidimarina NCIMB400. *Biochemistry* 38(11)**,** 3302-3309. doi: 10.1021/bi9826308.

Vickers, T.J., Greig, N., and Fairlamb, A.H. (2004). A trypanothione-dependent glyoxalase I with a prokaryotic ancestry in Leishmania major. *Proc Natl Acad Sci U S A* 101(36)**,** 13186-13191. doi: 10.1073/pnas.0402918101.

Wagner, A.F., Frey, M., Neugebauer, F.A., Schäfer, W., and Knappe, J. (1992). The free radical in pyruvate formate-lyase is located on glycine-734. *Proc Natl Acad Sci U S A* 89(3)**,** 996-1000. doi: 10.1073/pnas.89.3.996.

Wang, P., and Heitman, J. (2005). The cyclophilins. *Genome Biol* 6(7)**,** 226. doi: 10.1186/gb-2005-6-7-226.

Yang, Y., Harris, D.P., Luo, F., Wu, L., Parsons, A.B., Palumbo, A.V., et al. (2008). Characterization of the Shewanella oneidensis Fur gene: roles in iron and acid tolerance response. *BMC Genomics* 9 Suppl 1(Suppl 1)**,** S11. doi: 10.1186/1471-2164-9-s1-s11.

Zgurskaya, H.I., Krishnamoorthy, G., Ntreh, A., and Lu, S. (2011). Mechanism and Function of the Outer Membrane Channel TolC in Multidrug Resistance and Physiology of Enterobacteria. *Front Microbiol* 2**,** 189. doi: 10.3389/fmicb.2011.00189.

Zolkiewski, M. (1999). ClpB cooperates with DnaK, DnaJ, and GrpE in suppressing protein aggregation. A novel multi-chaperone system from Escherichia coli. *J Biol Chem* 274(40)**,** 28083-28086. doi: 10.1074/jbc.274.40.28083.
